# A Global Decline in Adolescents’ Subjective Well-Being: a Comparative Study Exploring Patterns of Change in the Life Satisfaction of 15-Year-Old Students in 46 Countries

**DOI:** 10.1007/s12187-020-09788-8

**Published:** 2020-12-15

**Authors:** Jose Marquez, Emily Long

**Affiliations:** 1 Faculty of Education, University of Cambridge, 184 Hills Rd, Cambridge CB2 8PQ, UK; 2 University of Glasgow, 99 Berkeley Square, Glasgow G3 7HR, UK

**Keywords:** Subjective well-being, Life satisfaction, PISA, Adolescence, Happiness

## Abstract

There is a growing body of research that demonstrates declines in subjective well-being and increases in mental health problems among children and young people in recent decades. However, there is little comparative research examining changes in adolescents’ life satisfaction (LS) across a large number of countries, and critically, how this differs across sociodemographic groups. This study addresses this question by investigating changes in the LS of 15-year-old students between 2015 and 2018, with particular attention given to differences by gender, socio-economic status, immigrant background and urbanity. Data for this study come from the Programme for International Student Assessment (PISA). Due to the skewed nature of LS scale variables, the current study includes both mean levels of LS in a 0 to 10 scale, and the proportion of students reporting low LS (5 points or less). Linear regression models were used. Results demonstrate a global decline in mean levels of LS in 39 out of the 46 countries. In most countries, mean LS declined more among girls than among boys. Mean LS declined more, and the proportion of students reporting low LS increased more, among non-immigrant students and those of higher SES in the majority of countries. Findings regarding rural or urban communities were mixed. We advise that heterogeneity across all sociodemographic groups needs to be accounted for in public policy efforts to increase LS among young people.

## 1 Introduction

Interest in measuring and monitoring progress in subjective well-being (SWB) has increased substantially in the last few decades. SWB refers to a person’s cognitive and affective evaluations of his or her life (Diener et al. 2002). Since the Stiglitz Commission (Stiglitz 2009) recommended that social progress should be assessed using SWB indicators, efforts to promote this have increased worldwide (Diener et al. 2018). Similarly, National governments acknowledge the need to measure SWB. For example, in the United Kingdom (UK), the Office for National Statistics has created a programme to measure national well-being for adults (ONS 2016), young people (ONS 2014a) and children (ONS 2014b), including subjective measures of well-being.

Interest in measuring and evaluating children’s SWB has traditionally attracted less attention, probably due to the lack of political importance attributed to children’s perspectives (Casas 2011). However, this has started to change and great progress has been made recently, particularly over the last decade. At the international level, together with the Health Behaviour in School-aged Children (HBSC) study (Currie 2012), which has been collecting data on children’s SWB for more than 3 decades, new studies collecting child SWB data have emerged. Most notably, this includes Children’s Worlds (Rees and Main 2015) and the Programme for International Student Assessment (PISA) by the Organisation for Economic Co-operation and Development (OECD) (OECD 2017a). This represents a substantial improvement in data available on children’s SWB internationally. This study takes advantage of the increasing data available at the international level to study changes in adolescents’ SWB across-countries. In particular, we use data from PISA 2015 and 2018 to study changes in the SWB (life satisfaction (LS)) of 15-year-old students in 46 countries.

### 1.1 Trends in Adolescents’ Subjective Well-Being and Related Outcomes

There is general academic consensus that child and adolescent well-being is a multidimensional concept that aims to measure the quality of children and adolescents’ lives (OECD 2009). Measures of subjective/hedonic well-being, eudaimonic/psychological well-being and mental health outcomes are commonly included within the construct of well-being (The Children’s Society 2019; Seligman 1999; Diener et al. 2018). Although there is not a complete overlap between well-being and mental ill-health, research shows that these are related (The Children’s Society 2019), and a robust evidence base demonstrates changes in these measures among children and young people in the last 20 years. For example, Twenge et al. (2018) examined changes in the psychological well-being of 8th, 10th and 12th graders between 1991 and 2016 in the United States (US) and found that adolescents’ self-esteem, LS and happiness suddenly decreased after 2012. Likewise, Twenge et al. (2017) found that, between 2010 and 2015, adolescents’ depressive symptoms, suicide-related outcomes, and suicide rates increased in the US, particularly among girls. These results are in line with those observed in other studies in the US, which report an increase in depression, nonfatal self-inflicted injury and suicide, particularly among girls, with upward trends starting around 2005–2008 (Curtin et al. 2016, Mercado et al. 2017, Mojtabai et al. 2016). Similarly, an increase in poor well-being and mental ill-health among adolescents has also been identified in different studies in the UK (Earle 2016; Patalay and Fitzsimons 2017; Frith 2016). McManus et al. (2019) demonstrated that non-suicidal self-harm, which is increasingly reported as a way of coping with unpleasant feelings of anger, tension, anxiety, or depression, almost tripled in England between 2000 and 2014, with the greatest increase being observed among women aged 16–24. Similar findings in relation to adolescents and young people’s SWB and mental health have been found in countries such as Germany, Sweden, Finland, Norway and New Zealand (Brann et al. 2017; Fleming et al. 2014; Brailovskaia and Margraf 2020; Bor et al. 2014; Kim and Hagquist 2018; Mishina et al. 2018; Potrebny et al. 2017, 2019).

### 1.2 Life Satisfaction

LS is a cognitive appraisal of one’s overall quality of life with a positive orientation (Huebner 2004). In research on children and adolescents, LS is the most common indicator used by researchers to study SWB (Proctor et al. 2009). Using HBSC data from 2002 and 2014, Due et al. (2019) explored changes in high life satisfaction (scoring 9 or 10 in the 0–10 scale) in 5 Nordic countries, finding contrasting trajectories: a decline in Denmark, Finland and Sweden, an increase in Norway and a less clear trend in Iceland. Cosma et al. (2020) used data from the last 5 HBSC studies (2002–2018) to explore changes in the mental well-being of 11–15-year-old adolescents in 36 countries, finding an increase in psychosomatic complaints in most of them but no overall changes in LS, which declined in 13 countries and increased in other 13. By contrast, The Good Childhood Report 2020 (The Children’s Society 2020) analysed data from Understanding Society in the UK, finding that the LS of 10–15-year-old children’s has been declining for almost a decade –especially among girls- and fell to its lowest level in the most recent wave of this survey (2017/18). Across multiple domains, this study indicated no variation in satisfaction with family, a sustained decline in satisfaction with friends since 2009, and a decline in satisfaction with appearance, schoolwork and school since 2015. This report also analysed data from PISA 2015 and 2018 on the LS of 15-year-old adolescents, finding that, across 24 European countries, adolescents in the UK reported the lowest levels of LS. Furthermore, between 2015 and 2018, LS declined in 19 out of 21 European countries and the largest declined was observed in the UK.

Despite evidence of negative trends in children and young people’s well-being and mental ill-health as a whole, and mixed evidence on trends in LS across multiple countries, scarce research has examined trends in adolescents’ LS across sociodemographic groups, and no study has explored this in a large number of countries. Thus, to fill this research gap, this study investigates changes in adolescents’ LS across a large number of countries. In particular, we use data from PISA 2015 and 2018 to study changes in the LS of 15-year-old students in 46 countries.

To accomplish this, we asked the following research questions:

1. Did the life satisfaction of 15-year-old adolescents increase, decrease or remain stable in the period 2015–2018?
2. Did changes in LS differ across gender, SES, immigrant background, rural/urban communities, or across countries?
3. Are patterns of change in LS across these groups similar or different when comparing variation in mean LS and the proportion of students reporting low LS?

## 2 Methods and Data

### 2.1 Pisa 2015, 2018

The current study uses data from PISA 2015 and PISA 2018 (*n* = 643,615; n (2015) = 312,028; n(2018) = 331,587). PISA is a worldwide study by the OECD, conducted in member and non-member countries and economies, carried out every 3 years and focused on 15-year-old students’ academic performance. PISA also collects a large amount of data on education policy and practice and, especially since 2015, on the broader well-being of students. More than 70 countries and economies participated in the 2015 and 2018 PISA editions, but data on LS was not collected in all countries. This study compares levels of students’ LS in the 46 countries and economies where LS data was collected and levels of missing data are low (see section 2.3). For ease of reading, we refer to all of them as countries regardless of the status of Hong Kong, Macao and Taiwan.

### 2.2 Variables

#### Life Satisfaction

In its 2015 edition, PISA introduced a framework to assess students’ well-being in several domains, including LS (Borgonovi and Pal 2016). In this study, LS is examined using the original PISA variable, which assesses LS using Cantril’s ladder (Cantril 1965) where participants are asked to rate how satisfied they feel about their lives these days from 0 (not at all satisfied) to 10 (completely satisfied). To facilitate the interpretation of the results reported in this study, it is important to clarify that mean changes of 0.3 or 0.5 on a 0–10 LS scale are considered as very large and are often found only in for major life events affecting the individual (OECD 2013). Furthermore, LS scales are positively skewed, as shown in Figs. A1.1 to A1.5 in Appendix 1. Consequently, mean levels of LS and the percentage of the population reporting low LS, defined as a LS rating of 5 or less, are examined.

#### Cohort

We study changes in LS using a dichotomous variable ‘cohort’, where 0 indicates that the respondent belongs to the 2015 data collection cohort and 1 to the 2018 data collection cohort.

#### Gender

Gender is measured by a binary variable that indicates whether the respondent is a girl (1) or a boy (2).

#### Socio-Economic Status

To investigate differences across SES, we created a categorical variable derived from PISA’s Economic, Social and Cultural Status (ESCS) index. First, we standardised this index with reference to each country such that 0 indicates the mean ESCS in each country and 1 the standard deviation. Then, from each country-specific continuous ESCS variable, we created the country-specific categorical SES variable, which indicates whether the student belongs to the bottom 25% in the country-specific ESCS scale (1), to the 50% in the middle (2) or to the top 25% (3).

#### Immigrant Background

To study differences by immigrant background, we created a dichotomous variable (non-immigrant, immigrant) reporting whether the student has an immigrant background or not. Non-immigrant students are those born in the country and whose parents were born in the country. Immigrant students are those who have an immigrant background, this is those born out of the country or whose father or mother was born out of the country.

#### Urban/Rural

To explore differences according to rural/urban location, we used PISA’s urbanity variable, which represents 5 levels on the rural-urban spectrum. In particular, this variable is derived from school principal’s responses to the following question: “Which of the following definitions best describes the community in which your school is located? A village, hamlet or rural area (fewer than 3000 people); A small town (3000 to about 15000 people); a town (15000 to about 100000 people); a city (100000 to about 1000000 people); a large city (with over 1000000 people)”.

#### Country

Finally, to study differences across the 46 countries, we included an additional country-level variable to identify participants from England, Wales, Northern Ireland and Scotland within the UK.

### 2.3 Analysis

The aim of the current study was to compare 2015 and 2018 cohorts of 15-year-old students in 46 countries to explore differences in LS and how these differ by gender, SES, immigrant background, urbanity and across countries. Linear regression techniques were used to assess whether LS increased, decreased or remain stable in this period, and to identify patterns of change across different groups of students. Our outcome variables were LS (0 to 10 scale, treated as a continuous variable) and the proportion of students reporting low LS (dichotomous variable). We used a single predictor variable –year, with a value of 0 for those who participated in 2015 and 1 for those who did it in 2018. We ran these models separately for each group of interest.

First, we examined changes in LS for the general population of 15-year-old students. The focus was on mean levels of LS and the proportion of students reporting low LS (i.e. rating their LS with 5 or less in the 0 to 10 LS scale). Then, we explored how mean levels of LS and the proportion of students reporting low LS had changed for girls and boys; for students of low, mid and high SES; for non-immigrant students and those with an immigrant background; and students attending schools located in more rural/ urban communities. Besides, we then explored differences across countries.

Final student weights were applied in the analysis to account for PISA’s complex design. Levels of missing data in the variables studied are low, below 10% in most countries. For those categories where levels of missing data are high (above 20%), these are excluded from the analysis. To avoid problems associated with small sample size, categories with less than 50 observations were excluded from the analysis. The analysis was conducted using Stata 15 (StataCorp 2017).

## 3 Results

### 3.1 Change in Students’ Life Satisfaction between 2015 and 2018

Table 1 reports changes in students’ LS between 2015 and 2018 in all the countries studied. As in all the other tables included in the following sections, it reports changes in mean levels of LS and the proportion of students reporting low LS. Results show that in Eastern Asian societies and Turkey, mean levels of LS were the lowest and the proportion of students reporting low LS the greatest. By contrast, in Mexico and Costa Rica, mean levels of LS are the highest and these were also the nations, together with the Netherlands and Finland, where the proportion of students reporting low LS was the smallest. Compared to these countries, the Netherlands had a lower mean LS, though a smaller proportion of students reported low LS. Indeed, the relative position of some countries in these two rankings (mean levels in LS and the proportion of students reporting low LS) changed substantially in some cases, suggesting that the variable LS (i.e. the 0 to 10 LS scale) was more skewed in some countries than in others. This is further supported by figs. A1.1 to A1.5 in Appendix 1, which show, for each country, the distribution of students’ responses in the LS scale used in PISA 2015 and 2018. Thus, focusing on both the mean levels of LS and the tail of the distribution (i.e. the proportion of students reporting low LS) is important when studying absolute levels and variation in students’ LS in PISA.

Table 1 also shows the existence of a global decline in the LS of 15-year-old students. Mean levels of LS declined in 39 out of 46 countries between 2015 and 2018. Similarly, the proportion of students reporting low LS increased in this period in most countries. Students’ mean levels of LS worsened the most in the UK, Japan, United States and Ireland, and also in France when considering the proportion of students reporting low LS.

In addition, in many countries the results demonstrated much greater variation in mean levels of LS than in the proportion of students reporting low LS, and vice versa. This suggests that there are cross-country differences in patterns of variation in LS, as changes in LS were more concentrated around the tail of the 0 to 10 LS scale in some countries than in others.

### 3.2 Gender Differences in Changes in Students’ Life Satisfaction between 2015 and 2018

Table 2 shows gender differences in changes in students’ LS between 2015 and 2018. In almost all countries, girls reported lower LS on average and the proportion of girls who reported low LS was greater than the proportion of boys who reported low LS.

Table 2 also shows that changes in LS between 2015 and 2018 tended to be more negative among girls than among boys. The decline in mean levels of LS was more accentuated among boys than among girls in 12 countries, while the opposite is observed in 31 countries. Among these, the decline is observed only among girls, but not among boys in 7 countries. Gender differences in the proportion of students reporting low LS appear less accentuated. In 19 countries, the increase was greater among girls than among boys, while the opposite is observed in 21 countries. Interestingly, for South Korea, Table 1 demonstrates an overall increase in students LS (0.15 points on average), but Table 2 reveals that this increase seems to hide important gender differences, as LS declined by -0.14 points, on average, among girls and increased by 0.42 points, on average, among boys. Likewise, in South Korea, the proportion of students who reported low LS increased among girls and decreased among boys.

### 3.3 Change in Students’ Life Satisfaction between 2015 and 2018: Differences across SES

Tables 3 and 4 illustrate SES differences in changes in students’ LS between 2015 and 2018. The comparison is between those in the bottom 25% of the SES distribution, those in the top 25% and the 50% in the middle. With a few exceptions (mainly in some Latin American countries), LS was on average lower, and the proportion of students reporting low LS greater, among students of lower SES than among students of higher SES.

Tables 3 and 4 show that in most countries, between 2015 and 2018, LS declined on average more among students of higher SES than among those of lower SES, although the opposite is observed in some countries like the UK. This pattern of change across SES is clearer when considering changes in the proportion of students who reported low LS. In almost all countries, the proportion of students who reported low LS increased more among students of higher SES than among those of lower SES. The results reported in Tables 3 and 4 reveal again the existence of cross-country differences in patterns of change in LS. This is, in some countries, changes were more concentrated in the tail of the 0 to 10 LS scale among students of lower SES while the opposite is observed in others.

### 3.4 Change in Students’ Life Satisfaction between 2015 and 2018: Differences by Immigrant Background

Differences in changes in students’ LS between 2015 and 2018 by immigrant background are reported in Table 5. Due to the small sample size (i.e., less than 50 observations) affecting some categories, results are not reported for comparisons involving these. Overall, in most countries, LS was on average higher, and the proportion of students reporting low LS smaller, among non-immigrant students than among students with an immigrant background, although exceptions to this were found.

Furthermore, Table 5 shows that the changes in LS in the period 2015–2018 were more negative among non-immigrant students than among students with an immigrant background. In particular, the decline in mean levels of LS was more accentuated among non-immigrant students than among students with an immigrant background in 21 countries, whereas the opposite was observed in 12 countries. The analysis of changes in the proportion of students reporting low LS revealed a clearer pattern of differences by immigrant background. In 24 countries, the increase in the proportion of students reporting low LS increased more among non-immigrant students than among students with an immigrant background, whereas the opposite is observed in 6 countries only. In Italy, LS declined among non-immigrant students, but no significant change among students with an immigrant background was found. Finally, differences across countries in the magnitude of the worsening in students’ LS were substantial.

### 3.5 Rural/Urban Differences in Changes in Students’ Life Satisfaction between 2015 and 2018

Differences in changes to students’ LS between 2015 and 2018 across levels of urbanity are reported in Tables 6 and 7. Again, results are not reported for comparisons involving categories with a small number of observations. Overall, in most countries, students’ LS was on average higher, and, although to a lesser extent, the proportion of students reporting low LS smaller, in rural communities compared to urban communities. However, there is great heterogeneity in the results and some notable exceptions to this observation.

Tables 6 and 7 show even more heterogeneity with regard to changes in LS between 2015 and 2018, and it is difficult to identify a pattern of change in most countries. For example, the results demonstrate greater declines in LS in urban communities in Japan, Turkey, Colombia, Bulgaria, while rural areas experienced greater declines for the UK as a whole, Qatar, Uruguay and Peru. In terms of the proportion of students reporting low LS, results are similar to those referring to mean levels of LS in some countries (e.g. UK, Qatar; Turkey, the Netherlands, Bulgaria, Austria, Ireland) but show different trajectories in others (Japan, Colombia; Uruguay, Peru). That is, in some countries (e.g., Turkey) the largest changes to both mean LS and proportion of students with low LS was found in urban areas, while other countries (e.g., Japan) demonstrated greater decreases to mean LS in urban areas, yet greater increases in the proportion of students with low LS in rural areas.

## 4 Discussion

The increasing policy interest in assessing children and young people’s well-being (ONS 2014a, 2014b) seems well justified in view of the growing body of research reporting declining levels of SWB and positive mental health (e.g. Twenge et al. 2017, 2018, The Children’s Society 2020, Earle 2016, Firth 2016, Mishina et al. 2018, Due et al. 2019). However, only a few studies have focused on changes in adolescents’ LS over time, presenting some contrasting results. Moreover, comparatively little research has examined changes in adolescent’s LS with regard to sociodemographic groups and how this differs across countries. Thus, the current study used a large data resource with 46 countries to investigate changes in LS between 2015 and 2018 according to gender, SES, immigration background, and urban/rural location.

Overall, the study found strong evidence that mean levels of LS declined in the majority of countries, and similarly, that the proportion of students reporting low LS increased in this period in most countries. This finding conflicts previous research that found mixed patterns in changes to LS (Due et al. 2019, Cosma et al. 2020), however, these studies considered a longer period of time (2002–2018), thus suggesting that the global decline in adolescents’ LS may be a relatively recent phenomenon. The Good Childhood Report 2020 showed that adolescents’ LS has been declining in the UK since 2011/2012 approximately (The Children’s Society 2020), but the moment in time in which declining levels in LS started may well differ across countries.

In addition, this study found important heterogeneity between countries and across the sociodemographic groups. In terms of gender, overall changes in LS between 2015 and 2018 were more negative among girls than among boys. Specifically, the decline in mean levels of LS was greater among girls in approximately 67% of the countries. However, gender differences in the proportion of students with low LS was less pronounced, with approximately 41% of the countries demonstrating a greater decline among girls, and approximately 44% indicating greater changes among boys. This suggests that although girls overall seemed to fare worse in LS between 2015 and 2018, in a non-negligible portion of countries, more boys than girls experienced more negative changes in their satisfaction with life. Moreover, Marquez (2020) revealed that, in 2015, in most countries, 15-year-old girls report lower LS than boys, and this study indicates that the gender gap, rather than reverting, is widening in many countries.

The study also found, overall, greater declines in LS among students of higher SES, than among those of lower SES. Similarly, the proportion of students who reported low LS increased more among students of higher SES than among those of lower SES in almost all countries. Although exceptions, such as the UK, were found, the study suggests counter-intuitive changes in LS related to SES. However, it is important to note that, in line with previous research (e.g. Marquez and Main 2020), LS was on average lower, and the proportion of students reporting low LS greater, among students of lower SES than among students of higher SES. As a result, the study suggests that students with low SES remained at relatively low levels of LS compared to their higher SES peers, while high SES students declined in LS overall.

Immigrant background influenced changes in LS such that greater decreases were found among non-immigrant students than among students with an immigrant background in most countries. In addition, non-immigrant students experienced a greater increase in the proportion of students reporting low LS compared to students with an immigrant background. Yet, similar to SES, LS was on average higher, and the proportion of students reporting low LS smaller, among non-immigrant students in the majority of countries. Thus, the study suggests that decreases in LS among non-immigrant students in recent years merits attention, yet these students still experience greater LS, on average, than students of an immigrant background.

Lastly, the study found inconclusive evidence on the relationship between urban/rural locality and trends in LS. Though the results demonstrated that, overall, students’ LS was on average higher in more rural communities than urban localities, evidence for changes to LS, both average changes and proportion of students with low LS, was mixed. Given the great heterogeneity of results, the study suggests that the association between urban/rural setting and LS varies according to the country of interest.

Previous research found some patterns across countries in correlates of adolescents’ LS (Rees and Main 2015, Marquez and Main 2020). However, these studies also reported great heterogeneity between countries and across groups. In relation to differences in trends in adolescents’ LS, the findings of this study are in line with these previous studies as we identified some general trends across countries together with a substantial level of heterogeneity between countries and groups. Furthermore, future research should explore what factors may be driving these negative trends in adolescents’ SWB and mental health outcomes, and in doing so, differences across countries and groups should be considered as well. Overall, this heterogeneity highlights the necessity of adopting a more nuanced approach when studying correlates and trends in adolescents’ SWB, as adolescents of different characteristics in different societies may have relatively distinct experiences that shape SWB in varied ways.

Heterogeneity across sociodemographic groups should also be considered in policy interventions intended to promote adolescents’ SWB and tackle these negative trends. For example, some interventions may need to be gender sensitive or target a particular group of students (e.g. immigrant students). Furthermore, the heterogeneity across countries, and in some countries, across rural/urban communities, suggests that policy efforts need to exercise caution when making generalisations across nations. Trends in LS observed in one society may well differ from those in a different one, or in a different region, state, or municipality. A policy response to deal with geographic heterogeneity in adolescents’ LS would be to promote the collection and analysis of data at the country level, but also, ideally, data which is representative of these different administrative levels and the rural/ urban population.

Finally, the current study highlights important differences between trends in mean LS and low LS, which was substantial across some countries and sociodemographic groups. Changes in different places on the LS scale (i.e., changes to mean levels of LS versus % with low LS) imply different at-risk groups. Consequently, policy efforts must delineate if they aim to improve average LS, or target interventions to those with the lowest LS scores, as these groups of adolescents may differ. In addition, future research should explore what changes in LS taking place in different part of the scale may mean in terms of other important outcomes, such as mental health outcomes. A better understanding of the interconnections between changes to LS and changes in SWB or mental health could inform policy efforts intended to promote better well-being overall.

## 5 Limitations

This study was affected by a series of limitations that are worth mentioning and which, ideally, should be addressed in future research. First, we focused on only one outcome variable (LS, derived from a single item scale from 0 to 10) and the study of other SWB measures may well provide results which differ from those reported in this study. Second, we focus on 15–16-year-old adolescents mainly in high-income countries who were enrolled in formal education. This is a rather restrictive definition of adolescence and, ideally, future research should explore this question for other age groups, for adolescents in and out of school, and in a wider variety of countries. Moreover, exclusions rates in PISA can be high in some countries (see Anders et al. 2020, OECD 2017b) and, for example, they exclude students with special needs. Furthermore, the study of gender differences was limited to a binary variable (girl/boy) and future research should aim at exploring this question for other gender identities. Finally, we focus on changes in a relatively short period of time –i.e. 3 years- and although results seem quite robust, future research should explore changes in a longer time frame.

## 6 Conclusions

There is an increasing body of research demonstrating declines in levels of well-being and positive mental health among adolescents in multiple countries, but little research has explored changes in LS and how these differ between countries and across groups. This study investigated this question, finding a rather global decline in the LS of 15-year-old adolescents between 2015 and 2018. In addition, despite general trends in LS, differences across countries and groups were important. In most countries, LS worsened more among girls, among adolescents of higher SES, and those without an immigrant background. Changes to LS relative to urban/rural location were mixed. Moreover, in some cases, changes in LS was more concentrated in the lower part of the 0 to 10 LS scale than in others, revealing further differences across countries and groups. Overall, this research highlights the necessity of adopting more nuanced approaches to studying trajectories in adolescents SWB, which may help us improve our understanding of adolescents well-being and strategies to promote it.

## Supplementary Material

Appendix

## Figures and Tables

**Table 1:** Change in students’ life satisfaction between 2015 and 2018

	Mean LS				% low LS				
	2015	2018	Change		2015	2018	Absolute change		Relative change
			B	S.E.			B	S.E.	
*Scotland*	*7.17*	*6.25*	−0.92***	*0.07*	*21.29%*	*32.43%*	11.14%***	*0.01*	*52.32%*
*England*	*6.94*	*6.12*	−0.82***	*0.06*	*24.05%*	*34.11%*	10.07%***	*0.01*	*41.86%*
United Kingdom	6.98	6.16	−0.81***	0.05	23.62%	33.66%	10.04%***	0.01	42.52%
*Wales*	*7.13*	*6.45*	−0.68***	*0.08*	*22.32%*	*30.61%*	8.29%***	*0.01*	*37.16%*
*Northern Ireland*	*7.24*	*6.58*	−0.67***	*0.08*	*20.18%*	*28.74%*	8.57%***	*0.01*	*42.45%*
Japan	6.80	6.18	−0.62***	0.05	27.12%	37.24%	10.12%***	0.01	37.30%
United States	7.36	6.75	−0.60***	0.05	20.01%	28.12%	8.11%***	0.01	40.52%
Ireland	7.30	6.74	−0.57***	0.06	18.66%	27.24%	8.58%***	0.01	45.98%
Qatar	7.41	6.84	−0.56***	0.04	21.84%	27.11%	5.26%***	0.01	24.10%
Brazil	7.59	7.05	−0.53***	0.05	17.65%	23.60%	5.95%***	0.01	33.72%
Macao	6.59	6.07	−0.52***	0.05	29.26%	36.43%	7.17%***	0.01	24.51%
Turkey	6.12	5.62	−0.50***	0.08	40.92%	45.46%	4.54%***	0.01	11.09%
Iceland	7.80	7.34	−0.46***	0.06	14.73%	17.51%	2.78%***	0.01	18.91%
France	7.63	7.19	−0.44***	0.04	13.84%	19.51%	5.67%***	0.01	40.99%
Poland	7.18	6.74	−0.44***	0.05	21.83%	27.37%	5.54%***	0.01	25.36%
Russia	7.76	7.32	−0.44***	0.07	16.78%	22.61%	5.83%***	0.01	34.76%
United Arab Emirates	7.30	6.88	−0.42***	0.05	23.80%	27.92%	4.11%***	0.01	17.28%
Austria	7.52	7.14	−0.39***	0.06	17.05%	22.20%	5.15%***	0.01	30.21%
Luxembourg	7.38	7.04	−0.34***	0.05	18.81%	23.37%	4.55%***	0.01	24.20%
Switzerland	7.72	7.38	−0.34***	0.05	12.79%	16.97%	4.18%***	0.01	32.67%
Chile	7.37	7.03	−0.34***	0.06	21.44%	25.29%	3.85%***	0.01	17.93%
Germany	7.35	7.02	−0.33***	0.05	16.40%	20.46%	4.06%***	0.01	24.73%
Netherlands	7.83	7.50	−0.33***	0.04	6.72%	8.49%	1.77%***	0.01	26.34%
Slovenia	7.17	6.86	−0.32***	0.06	21.81%	26.33%	4.52%***	0.01	20.75%
Estonia	7.50	7.19	−0.31***	0.05	17.52%	20.89%	3.38%***	0.01	19.28%
Finland	7.89	7.61	−0.28***	0.05	11.26%	15.14%	3.88%***	0.01	34.45%
Colombia	7.88	7.62	−0.27***	0.05	16.51%	18.62%	2.11%***	0.01	12.75%
Bulgaria	7.42	7.15	−0.26***	0.05	21.22%	23.27%	2.05%***	0.01	9.64%
Lithuania	7.86	7.61	−0.26***	0.05	14.43%	17.66%	3.24%***	0.01	22.43%
Slovakia	7.47	7.22	−0.25***	0.05	19.33%	21.70%	2.37%***	0.01	12.25%
Costa Rica	8.21	7.96	−0.25***	0.05	11.11%	14.34%	3.23%***	0.01	29.10%
Portugal	7.36	7.13	−0.24***	0.05	17.53%	20.15%	2.61%***	0.01	14.92%
Croatia	7.90	7.69	−0.22***	0.04	12.89%	17.02%	4.13%***	0.01	32.02%
Latvia	7.37	7.16	−0.21***	0.05	16.97%	21.14%	4.17%***	0.01	24.55%
Hong Kong	6.48	6.27	−0.20***	0.05	28.15%	30.91%	2.75%***	0.01	9.78%
Peru	7.50	7.31	−0.19***	0.05	20.98%	19.76%	−1.22%***	0.01	−5.82%
Uruguay	7.70	7.54	−0.16**	0.06	16.81%	17.29%	0.48%***	0.01	2.83%
Mexico	8.27	8.11	−0.16***	0.05	11.67%	11.10%	−0.57%***	0.01	−4.88%
Czech Republic	7.05	6.91	−0.14*	0.06	24.13%	25.21%	1.08%***	0.01	4.47%
Thailand	7.71	7.64	−0.08	0.05	17.67%	19.89%	2.23%***	0.01	12.60%
Taiwan	6.59	6.52	−0.07	0.05	31.48%	32.14%	0.65%***	0.01	2.08%
Spain	7.42	7.35	−0.07	0.04	16.09%	17.23%	1.14%***	0.01	7.11%
Montenegro	7.75	7.69	−0.06	0.05	18.12%	19.75%	1.63%***	0.01	8.98%
Hungary	7.17	7.12	−0.06	0.07	21.61%	22.69%	1.09%***	0.01	5.04%
Italy	6.89	6.91	0.02	0.06	24.11%	22.32%	−1.79%***	0.01	−7.42%
Greece	6.91	6.99	0.07	0.05	24.09%	23.09%	−1.00%***	0.01	−4.16%
South Korea	6.36	6.52	0.15*	0.06	34.19%	32.58%	−1.60%***	0.01	−4.69%

Notes: countries are ordered from greater to smaller change in mean life satisfaction in the period 2015-2018 Low life satisfaction is defined as rating your life satisfaction with 5 or less in the 0 to 10 life satisfaction scale.

* indicates *p* <.05, ** *p* <.01, and *** *p* <.001

**Table 2:** Change in students’ life satisfaction between 2015 and 2018, by gender

	Mean LS								% low LS									
	2015		2018		Change				2015		2018		Absolute change				Relative change	
	Girls	Boys	Girls	Boys	Girls		Boys		Girls	Boys	Girls	Boys	Girls		Boys		Girls	Boys
					B	S.E.	B	S.E.					B	S.E.	B	S.E.		
*England*	*6.61*	*7.26*	*5.70*	*6.59*	*−0.91* ***	*0.08*	*−0.68* ***	*0.07*	28.81%	19.42%	41.41%	26.32%	12.59%***	*0.01*	6.89***	*0.01*	43.71%	35.48%
United Kingdom	6.64	7.31	5.76	6.61	−0.88***	0.07	−0.71***	0.06	28.54%	18.85%	40.75%	26.15%	12.21%***	0.01	7.30%***	0.01	42.78%	38.74%
*Scotland*	*6.73*	*7.60*	*5.96*	*6.56*	−0.76***	*0.10*	−1.04***	*0.09*	27.76%	15.05%	38.08%	26.65%	10.32%***	*0.02*	11.60%***	*0.02*	37.19%	77.04%
Brazil	7.45	7.74	6.73	7.40	−0.72***	0.07	−0.34***	0.06	19.76%	15.42%	27.76%	19.45%	8.00%***	0.01	4.03%***	0.01	40.47%	26.15%
Japan	6.86	6.74	6.18	6.18	−0.68***	0.07	−0.56***	0.07	26.22%	28.02%	37.24%	37.25%	11.02%***	0.01	9.23%***	0.01	42.03%	32.94%
*Northern Ireland*	*6.94*	*7.54*	*6.27*	*6.90*	−0.67***	*0.12*	−0.64***	*0.11*	24.22%	16.19%	33.48%	23.95%	9.26%***	*0.02*	7.77%***	*0.02*	38.22%	47.97%
*Wales*	*6.74*	*7.52*	*6.07*	*6.84*	−0.67***	*0.11*	−0.68***	*0.11*	27.97%	16.91%	37.53%	23.80%	9.56%***	*0.02*	6.89%***	*0.02*	34.18%	40.71%
Macao	6.59	6.60	5.93	6.22	−0.66***	0.06	−0.38***	0.07	28.53%	29.98%	39.23%	33.71%	10.70%***	0.01	3.72%**	0.01	37.50%	12.42%
United States	7.06	7.66	6.47	7.03	−0.59***	0.06	−0.63***	0.08	24.52%	15.51%	32.82%	23.63%	8.30%***	0.01	8.12%***	0.01	33.84%	52.34%
Qatar	7.30	7.51	6.73	6.97	−0.57***	0.05	−0.54***	0.05	24.70%	19.12%	30.71%	23.61%	6.01%***	0.01	4.49%***	0.01	24.33%	23.48%
Ireland	7.02	7.58	6.45	7.02	−0.57***	0.07	−0.56***	0.07	23.17%	14.38%	31.44%	23.00%	8.27%***	0.01	8.62%***	0.01	35.67%	59.93%
Poland	6.83	7.53	6.30	7.19	−0.53***	0.08	−0.33***	0.07	27.10%	16.75%	33.68%	21.05%	6.58%***	0.01	4.30%***	0.01	24.27%	25.69%
Russia	7.60	7.92	7.08	7.57	−0.53***	0.08	−0.35***	0.08	19.28%	14.20%	26.51%	18.65%	7.24%***	0.01	4.44%***	0.01	37.54%	31.27%
United Arab Emirates	7.17	7.44	6.69	7.10	−0.48***	0.07	−0.34***	0.07	26.52%	21.01%	31.49%	24.24%	4.97%***	0.01	3.23%**	0.01	18.74%	15.40%
France	7.41	7.86	6.93	7.44	−0.47***	0.06	−0.42***	0.05	16.53%	11.10%	23.47%	15.66%	6.93%***	0.01	4.56%***	0.01	41.93%	41.06%
Iceland	7.35	8.28	6.94	7.76	−0.41***	0.07	−0.52***	0.08	20.12%	9.00%	22.31%	12.65%	2.18%	0.01	3.65%**	0.01	10.85%	40.60%
Estonia	7.27	7.73	6.87	7.52	−0.40***	0.07	−0.21***	0.06	20.99%	14.18%	25.11%	16.68%	4.13%**	0.01	2.50%**	0.01	19.66%	17.60%
Slovenia	6.71	7.62	6.30	7.40	−0.40***	0.09	−0.22**	0.07	28.91%	15.16%	33.55%	19.45%	4.64%**	0.02	4.30%***	0.01	16.04%	28.34%
Chile	7.13	7.60	6.73	7.32	−0.40***	0.08	−0.28***	0.08	25.73%	17.21%	29.67%	21.02%	3.94%**	0.01	3.82%**	0.01	15.32%	22.17%
Latvia	7.29	7.46	6.91	7.43	−0.39***	0.07	−0.02	0.07	18.15%	15.80%	24.39%	17.79%	6.24%***	0.01	1.99%	0.01	34.40%	12.56%
Netherlands	7.56	8.11	7.18	7.83	−0.37***	0.05	−0.28***	0.05	9.21%	4.21%	11.47%	5.52%	2.26%*	0.01	1.31%*	0.01	24.54%	31.15%
Costa Rica	8.04	8.39	7.67	8.25	−0.36***	0.07	−0.13*	0.06	12.92%	9.23%	17.53%	11.07%	4.61%***	0.01	1.84%*	0.01	35.65%	19.92%
Croatia	7.62	8.21	7.28	8.11	−0.34***	0.05	−0.10	0.06	16.35%	9.16%	22.13%	11.85%	5.77%***	0.01	2.69%***	0.01	35.29%	29.38%
Switzerland	7.38	8.03	7.04	7.68	−0.34***	0.07	−0.35***	0.07	17.08%	8.84%	21.45%	12.93%	4.37%***	0.01	4.09%***	0.01	25.62%	46.25%
Germany	6.96	7.76	6.62	7.37	−0.33***	0.06	−0.39***	0.07	21.89%	11.11%	25.56%	16.08%	3.67%**	0.01	4.96%***	0.01	16.79%	44.66%
Slovakia	7.17	7.76	6.85	7.59	−0.31***	0.07	−0.17*	0.08	23.49%	15.41%	25.89%	17.45%	2.40%	0.01	2.04%*	0.01	10.21%	13.21%
Austria	7.09	7.95	6.78	7.49	−0.31***	0.07	−0.46***	0.07	23.07%	11.15%	27.00%	17.55%	3.93%**	0.01	6.40%***	0.01	17.05%	57.42%
Finland	7.51	8.25	7.21	8.02	−0.30***	0.07	−0.23***	0.06	15.22%	7.57%	20.08%	10.39%	4.85%***	0.01	2.82%***	0.01	31.88%	37.26%
Turkey	5.83	6.41	5.53	5.71	−0.30*	0.12	−0.70***	0.09	45.59%	36.26%	47.80%	43.16%	2.21%	0.02	6.91%***	0.01	4.84%	19.05%
Peru	7.42	7.57	7.13	7.48	−0.30***	0.08	−0.09	0.07	22.28%	19.69%	21.32%	18.26%	−0.97%	0.01	−1.43%	0.01	−4.33%	−7.25%
Colombia	7.71	8.08	7.42	7.83	−0.29***	0.07	−0.25***	0.07	19.03%	13.71%	20.69%	16.46%	1.66%	0.01	2.75%**	0.01	8.74%	20.08%
Lithuania	7.60	8.12	7.33	7.88	−0.27***	0.06	−0.25***	0.07	17.27%	11.67%	21.31%	14.17%	4.04%***	0.01	2.51%**	0.01	23.36%	21.51%
Luxembourg	6.99	7.78	6.74	7.34	−0.26***	0.07	−0.44***	0.07	23.64%	13.92%	27.19%	19.65%	3.54%**	0.01	5.73%***	0.01	14.99%	41.20%
Uruguay	7.47	7.95	7.22	7.90	−0.25***	0.07	−0.05	0.08	19.64%	13.71%	21.01%	13.24%	1.37%	0.01	−0.46%	0.01	6.97%	−3.39%
Hong Kong	6.44	6.51	6.19	6.35	−0.25***	0.06	−0.16*	0.08	27.71%	28.59%	31.22%	30.61%	3.50%*	0.01	2.03%	0.02	12.65%	7.10%
Portugal	7.11	7.61	6.87	7.38	−0.23***	0.07	−0.24***	0.06	21.57%	13.57%	23.69%	16.71%	2.12%	0.01	3.14%**	0.01	9.83%	23.11%
Mexico	8.21	8.33	7.98	8.25	−0.23***	0.07	−0.07	0.06	13.04%	10.34%	12.49%	9.61%	−0.56%	0.01	−0.74%	0.01	−4.28%	−7.13%
Bulgaria	7.20	7.62	6.97	7.32	−0.22**	0.07	−0.29***	0.07	23.77%	18.94%	25.90%	20.94%	2.13%	0.01	2.00%	0.01	8.96%	10.56%
Taiwan	6.45	6.74	6.30	6.74	−0.15**	0.05	0.01	0.06	33.75%	29.26%	36.05%	28.26%	2.29%*	0.01	−1.00%	0.01	6.80%	−3.42%
Czech Republic	6.72	7.37	6.57	7.24	−0.15	0.09	−0.13	0.07	29.06%	19.46%	29.10%	21.49%	0.04%	0.02	2.03%	0.01	0.13%	10.43%
South Korea	6.12	6.59	5.98	7.01	−0.14*	0.07	0.42***	0.07	38.01%	30.69%	41.02%	24.92%	3.01%*	0.01	−5.76%***	0.01	7.91%	−18.78%
Thailand	7.70	7.73	7.56	7.72	−0.13	0.08	−0.01	0.06	17.04%	18.49%	19.96%	19.82%	2.92%*	0.01	1.32%	0.01	17.16%	7.16%
Spain	7.24	7.60	7.12	7.57	−0.11*	0.05	−0.04	0.05	18.71%	13.45%	20.10%	14.43%	1.39%	0.01	0.98%	0.01	7.41%	7.27%
Montenegro	7.50	7.99	7.42	7.95	−0.09	0.07	−0.04	0.07	20.99%	15.38%	23.78%	16.05%	2.79%*	0.01	0.67%	0.01	13.30%	4.33%
Hungary	6.80	7.54	6.72	7.52	−0.08	0.09	−0.02	0.08	27.10%	16.13%	27.97%	17.31%	0.88%	0.01	1.18%	0.01	3.23%	7.31%
Italy	6.50	7.29	6.54	7.27	0.04	0.08	−0.03	0.06	31.17%	16.96%	27.64%	17.36%	−3.53%**	0.01	0.41%	0.01	−11.34%	2.40%
Greece	6.59	7.22	6.74	7.24	0.15**	0.06	0.02	0.08	29.40%	19.19%	27.09%	19.20%	−2.32%*	0.01	0.01%	0.01	−7.88%	0.04%

Notes: countries are ordered from greater to smaller change in mean life satisfaction among girls in the period 2015–2018

Low life satisfaction is defined as rating your life satisfaction with 5 or less in the 0 to 10 life satisfaction scale.

* p < .05, ** p < .01, and *** p < .001

**Table 3:** Change in students’ mean life satisfaction between 2015 and 2018, by SES

	2015		2018				Change					
	Low	Mid	High	Low	Mid	High	Low		Mid		High	
							B	S.E.	B	S.E.	B	S.E.
Scotland	6.98	7.14	7.39	5.85	6.33	6.49	−1.12***	0.15	−0.81***	0.09	−0.90***	0.12
United Arab Emirates	7.03	7.24	7.72	6.95	6.88	6.83	−0.08	0.08	−0.36***	0.06	−0.89***	0.12
Qatar	7.16	7.37	7.72	6.76	6.83	6.94	−0.40***	0.07	−0.54***	0.05	−0.78***	0.06
United Kingdom	6.68	7.01	7.27	5.76	6.13	6.54	−0.92***	0.09	−0.88***	0.07	−0.73***	0.08
England	6.63	7.00	7.25	5.71	6.09	6.52	−0.91***	0.10	−0.91***	0.09	−0.72***	0.10
Japan	6.58	6.85	6.96	6.14	6.15	6.28	−0.43***	0.10	−0.70***	0.06	−0.67***	0.10
Ireland	7.26	7.26	7.44	6.57	6.76	6.82	−0.68***	0.10	−0.49***	0.09	−0.62***	0.08
Iceland	7.47	7.76	8.20	7.04	7.35	7.61	−0.43**	0.15	−0.40***	0.09	−0.60***	0.11
Northern Ireland	7.05	7.21	7.46	6.51	6.46	6.87	−0.54***	0.16	−0.75***	0.11	−0.59***	0.16
Wales	6.93	7.14	7.39	5.98	6.51	6.80	−0.94***	0.15	−0.63***	0.09	−0.58***	0.13
United States	7.00	7.37	7.66	6.39	6.74	7.08	−0.60***	0.11	−0.63***	0.07	−0.58***	0.10
Lithuania	7.61	7.82	8.21	7.53	7.62	7.66	−0.08	0.09	−0.20**	0.07	−0.55***	0.10
Macao	6.33	6.62	6.80	5.77	6.13	6.26	−0.56***	0.09	−0.49***	0.06	−0.54***	0.10
Brazil	7.73	7.53	7.56	7.12	7.04	7.04	−0.61***	0.09	−0.49***	0.06	−0.53***	0.08
Russia	7.71	7.70	7.93	7.38	7.23	7.43	−0.32**	0.11	−0.47***	0.07	−0.50***	0.10
Poland	6.88	7.40	7.36	6.52	7.07	6.87	−0.36***	0.10	−0.46***	0.08	−0.49***	0.11
France	7.40	7.62	7.89	6.89	7.19	7.45	−0.51***	0.10	−0.43***	0.06	−0.45***	0.07
Chile	7.11	7.41	7.76	6.76	7.10	7.32	−0.35**	0.11	−0.31***	0.07	−0.44***	0.10
Austria	7.25	7.56	7.75	7.07	7.09	7.31	−0.18	0.10	−0.47***	0.09	−0.44***	0.08
Turkey	5.96	6.11	6.28	5.33	5.63	5.87	−0.64***	0.14	−0.48***	0.11	−0.41**	0.13
Germany	7.08	7.38	7.57	6.87	7.01	7.19	−0.21*	0.11	−0.36***	0.07	−0.38***	0.10
Switzerland	7.65	7.67	7.88	7.32	7.32	7.52	−0.33**	0.11	−0.35***	0.07	−0.36***	0.08
Costa Rica	8.22	8.19	8.24	8.04	7.93	7.91	−0.18	0.09	−0.25***	0.06	−0.33***	0.08
Hong Kong	6.23	6.45	6.79	6.00	6.31	6.47	−0.23**	0.09	−0.14*	0.06	−0.32**	0.10
Estonia	7.18	7.50	7.88	6.96	7.13	7.58	−0.23*	0.10	−0.37***	0.07	−0.30**	0.10
Slovenia	7.20	7.15	7.21	6.84	6.83	6.91	−0.36**	0.13	−0.32***	0.08	−0.30*	0.12
Slovakia	7.19	7.53	7.62	7.01	7.25	7.35	−0.18	0.10	−0.28***	0.07	−0.28**	0.09
Bulgaria	7.11	7.45	7.65	6.91	7.19	7.38	−0.20	0.13	−0.27***	0.08	−0.27***	0.08
Colombia	8.02	7.85	7.77	7.79	7.59	7.49	−0.23*	0.10	−0.26***	0.07	−0.27**	0.09
Croatia	7.79	7.93	7.95	7.56	7.76	7.68	−0.24*	0.09	−0.17**	0.06	−0.27***	0.07
Hungary	6.86	7.16	7.55	6.91	7.15	7.28	0.04	0.12	−0.01	0.08	−0.27*	0.11
Netherlands	7.85	7.82	7.82	7.54	7.46	7.56	−0.31**	0.10	−0.36***	0.06	−0.26***	0.07
Peru	7.57	7.49	7.45	7.42	7.32	7.20	−0.15	0.12	−0.16*	0.07	−0.25**	0.09
Finland	7.69	7.86	8.15	7.26	7.65	7.89	−0.43***	0.09	−0.21***	0.05	−0.25***	0.06
Luxembourg	7.19	7.32	7.69	6.72	6.98	7.47	−0.48***	0.12	−0.34***	0.06	−0.22*	0.09
Latvia	7.11	7.32	7.71	6.76	7.17	7.53	−0.35**	0.12	−0.15*	0.07	−0.18*	0.09
Taiwan	6.32	6.61	6.82	6.27	6.59	6.64	−0.06	0.08	−0.03	0.06	−0.18*	0.08
Czech Republic	6.71	7.10	7.36	6.71	6.92	7.19	0.00	0.13	−0.19*	0.08	−0.17*	0.08
Spain	7.25	7.35	7.78	7.11	7.34	7.63	−0.14*	0.07	0.00	0.06	−0.14*	0.06
Mexico	8.22	8.25	8.37	7.92	8.15	8.23	−0.30**	0.11	−0.10	0.06	−0.13	0.07
South Korea	6.19	6.29	6.68	6.42	6.55	6.55	0.23*	0.09	0.26***	0.08	−0.12	0.09
Greece	6.67	6.96	7.10	6.92	7.01	7.02	0.25	0.13	0.05	0.06	−0.09	0.10
Portugal	7.18	7.37	7.44	7.00	6.83	7.37	−0.18	0.10	−0.33***	0.06	−0.07	0.09
Uruguay	7.48	7.69	7.94	7.20	7.53	7.90	−0.28*	0.13	−0.17*	0.08	−0.04	0.10
Thailand	7.79	7.72	7.55	7.65	7.65	7.56	−0.14	0.10	−0.06	0.05	0.01	0.11
Montenegro	7.59	7.81	7.78	7.49	7.74	7.79	−0.11	0.10	−0.07	0.06	0.02	0.11
Italy	6.70	6.90	7.08	6.71	6.89	7.14	0.01	0.11	−0.01	0.07	0.07	0.07

Notes: countries are ordered from greater to smaller change in mean life satisfaction among students of high SES in the period 2015–2018. Students of higher SES are those in the top 25% of the SES scale (PISA’s ESCS variable) in their country, students of low SES are those in the bottom 25% and the rest are considered students of mid SES

* p < .05, ** p < .01, and *** p < .001

**Table 4:** Change in the proportion of students reporting low life satisfaction between 2015 and 2018, by SES

	2015			2018			Absolute change						Relative change		
	Low	Mid	High	Low	Mid	High	Low		Mid		High		Low	Mid	High
							B	S.E.	B	S.E.	B	S.E.			
Scotland	25.93%	22.22%	16.74%	39.12%	32.51%	28.31%	13.18%***	0.02	10.29%***	0.02	11.57%***	0.02	50.83%	46.33%	69.10%
United Arab Emirates	29.75%	24.69%	17.51%	29.37%	28.38%	28.72%	−0.38%	0.01	3.69%***	0.01	11.21%***	0.02	−1.28%	14.94%	63.98%
Qatar	26.57%	22.52%	16.50%	29.23%	28.07%	25.67%	2.66%*	0.01	5.55%***	0.01	9.17%***	0.01	10.01%	24.66%	55.57%
United Kingdom	30.03%	23.52%	18.25%	40.69%	35.86%	28.50%	10.66%***	0.02	12.34%***	0.01	10.26%***	0.01	35.52%	52.48%	56.20%
England	30.68%	23.37%	18.50%	41.40%	36.50%	28.16%	10.72%***	0.02	13.13%***	0.01	9.66%***	0.02	34.93%	56.16%	52.22%
Japan	31.87%	25.58%	24.51%	39.73%	37.12%	35.50%	7.86%***	0.02	11.54%***	0.01	10.99%***	0.02	24.67%	45.10%	44.85%
Ireland	21.08%	19.33%	15.61%	31.00%	26.53%	25.48%	9.92%***	0.02	7.20%***	0.01	9.87%***	0.02	47.07%	37.27%	63.23%
Iceland	19.06%	15.30%	10.46%	21.66%	17.24%	15.20%	2.60%	0.02	1.94%	0.01	4.74%**	0.02	13.67%	12.68%	45.35%
Northern Ireland	26.10%	20.60%	16.09%	31.00%	30.71%	24.90%	4.90%	0.02	10.11%***	0.02	8.81%***	0.02	18.75%	49.10%	54.77%
Wales	27.29%	22.35%	18.33%	39.57%	31.40%	27.25%	12.28%***	0.03	9.05%***	0.01	8.92%***	0.02	45.00%	40.49%	48.64%
United States	27.76%	19.58%	14.39%	34.95%	28.48%	22.38%	7.18%***	0.02	8.89%***	0.01	7.99%***	0.02	25.87%	45.40%	55.52%
Lithuania	19.36%	14.71%	9.96%	19.69%	17.54%	16.55%	0.34%	0.01	2.83%**	0.01	6.59%***	0.01	1.74%	19.27%	66.22%
Macao	33.19%	29.36%	24.98%	41.56%	35.59%	32.81%	8.37%***	0.02	6.23%***	0.01	7.83%***	0.02	25.22%	21.23%	31.35%
Brazil	17.87%	19.17%	17.98%	21.85%	24.67%	24.52%	3.98%***	0.01	5.50%***	0.01	6.53%***	0.01	22.25%	28.70%	36.33%
Russia	18.82%	18.04%	14.78%	23.05%	23.86%	21.37%	4.24%*	0.02	5.82%***	0.01	6.59%***	0.01	22.50%	32.27%	44.57%
Poland	26.82%	20.87%	18.83%	30.69%	26.95%	25.34%	3.87%*	0.02	6.07%***	0.01	6.51%***	0.02	14.43%	29.09%	34.59%
France	18.58%	14.11%	8.90%	25.33%	19.54%	14.94%	6.75%***	0.02	5.43%***	0.01	6.04%***	0.01	36.30%	38.49%	67.91%
Chile	27.26%	20.41%	13.33%	30.78%	24.52%	19.98%	3.52%*	0.02	4.12%***	0.01	6.65%***	0.01	12.91%	20.17%	49.93%
Austria	22.65%	15.71%	13.95%	24.13%	22.64%	19.62%	1.48%	0.02	6.93%***	0.01	5.67%***	0.01	6.52%	44.14%	40.60%
Turkey	44.44%	41.86%	36.60%	51.12%	45.43%	41.04%	6.68%**	0.02	3.58%*	0.02	4.43%	0.02	15.02%	8.54%	12.11%
Germany	23.48%	18.34%	13.71%	25.65%	23.53%	20.58%	2.17%	0.02	5.18%***	0.01	6.87%***	0.02	9.26%	28.26%	50.14%
Switzerland	15.16%	12.61%	10.47%	18.87%	17.98%	13.83%	3.71%*	0.02	5.37%***	0.01	3.36%*	0.02	24.51%	42.56%	32.05%
Costa Rica	13.08%	11.09%	10.07%	14.08%	14.76%	14.08%	1.01%	0.01	3.67%***	0.01	4.01%***	0.01	7.69%	33.08%	39.86%
Hong Kong	34.41%	29.06%	21.97%	37.72%	30.82%	27.42%	3.32%	0.02	1.76%	0.01	5.45%*	0.02	9.64%	6.05%	24.80%
Estonia	23.70%	16.95%	12.58%	24.99%	22.07%	15.37%	1.28%	0.02	5.12%***	0.01	2.79%	0.02	5.41%	30.20%	22.16%
Slovenia	22.91%	22.38%	20.34%	26.29%	27.78%	24.88%	3.38%	0.02	5.40%***	0.01	4.54%*	0.02	14.76%	24.14%	22.33%
Slovakia	24.27%	18.63%	16.71%	24.62%	22.13%	18.76%	0.35%	0.02	3.50%**	0.01	2.05%	0.01	1.45%	18.78%	12.29%
Bulgaria	27.24%	20.95%	17.30%	27.20%	23.40%	20.42%	−0.04%	0.02	2.44%*	0.01	3.13%*	0.01	−0.15%	11.67%	18.09%
Colombia	16.60%	16.87%	16.88%	17.84%	20.10%	20.21%	1.25%	0.01	3.23%**	0.01	3.33%*	0.02	7.53%	19.16%	19.73%
Croatia	14.50%	13.14%	11.53%	18.60%	16.79%	16.22%	4.10%**	0.02	3.65%***	0.01	4.69%***	0.01	28.26%	27.76%	40.70%
Hungary	27.84%	22.02%	14.88%	26.50%	22.47%	19.51%	−1.34%	0.02	0.45%	0.01	4.63%**	0.02	−4.80%	2.03%	31.11%
Netherlands	7.82%	6.74%	5.70%	9.01%	9.08%	7.30%	1.18%	0.01	2.34%**	0.01	1.61%	0.01	15.14%	34.69%	28.20%
Peru	22.60%	21.13%	19.22%	16.72%	20.58%	21.85%	−5.88%***	0.01	−0.55%	0.01	2.63%	0.01	−26.03%	−2.61%	13.70%
Finland	14.91%	11.37%	7.71%	20.02%	14.83%	11.52%	5.11%***	0.01	3.45%***	0.01	3.81%***	0.01	34.28%	30.38%	49.39%
Luxembourg	23.43%	19.44%	13.38%	29.02%	24.24%	16.43%	5.59%**	0.02	4.80%***	0.01	3.05%*	0.01	23.88%	24.71%	22.77%
Latvia	21.08%	17.67%	12.08%	28.56%	20.83%	15.90%	7.48%***	0.02	3.16%**	0.01	3.82%**	0.01	35.48%	17.90%	31.60%
Taiwan	38.44%	30.49%	26.65%	37.44%	31.99%	28.20%	−1.00%	0.02	1.50%	0.01	1.55%	0.02	−2.59%	4.91%	5.83%
Czech Republic	30.90%	23.52%	17.97%	29.17%	25.31%	19.96%	−1.72%	0.02	1.79%	0.01	1.99%	0.01	−5.58%	7.62%	11.09%
Spain	18.94%	17.34%	10.14%	21.03%	17.38%	13.15%	2.10%	0.01	0.04%	0.01	3.00%**	0.01	11.06%	0.21%	29.63%
Mexico	14.73%	11.30%	9.25%	13.10%	12.63%	11.31%	−1.62%	0.02	1.34%	0.01	2.07%	0.01	−11.03%	11.82%	22.33%
South Korea	39.06%	34.66%	29.02%	34.54%	31.97%	32.10%	−4.52%**	0.02	−2.69%	0.01	3.07%	0.02	−11.58%	−7.76%	10.59%
Greece	29.42%	23.58%	19.66%	24.95%	22.63%	22.54%	−4.48%*	0.02	−0.95%	0.01	2.88%	0.02	−15.22%	−4.03%	14.66%
Portugal	23.04%	17.33%	14.81%	25.30%	21.68%	16.01%	2.26%	0.02	4.35%***	0.01	1.20%	0.01	9.83%	25.10%	8.12%
Uruguay	21.19%	17.34%	12.01%	22.54%	17.99%	13.00%	1.35%	0.02	0.65%	0.01	0.99%	0.02	6.39%	3.72%	8.28%
Thailand	18.79%	18.31%	15.89%	22.00%	19.91%	16.73%	3.22%*	0.02	1.60%	0.01	0.84%	0.02	17.13%	8.76%	5.30%
Montenegro	20.65%	17.72%	17.82%	22.47%	19.26%	18.50%	1.82%	0.02	1.55%	0.01	0.68%	0.02	8.82%	8.75%	3.81%
Italy	29.09%	23.98%	20.90%	25.33%	23.59%	18.57%	−3.76%*	0.02	−0.39%	0.01	−2.33%	0.01	−12.92%	−1.62%	−11.15%

Notes: countries are ordered from greater to smaller change in life satisfaction among students of high SES in the period 2015–2018. Students of high SES are those in the top 25% of the SES scale (PISA’s ESCS variable) in their country, students of low SES are those in the bottom 25% and the rest are considered students of mid SES

Low life satisfaction is defined as rating your life satisfaction with 5 or less in the 0 to 10 life satisfaction scale.

* p < .05, ** p < .01, and *** p < .001

**Table 5:** Change in students’ life satisfaction between 2015 and 2018, by immigrant background

	Mean LS								% low LS									
	2015		2018		Change				2015		2018		Absolute change				Relative change	
	Nat.	Imm.	Nat.	Imm.	Native		Immigrant		Native	Immig.	Native	Immig	Native		Immigrant		Native	Immig.
					B	S.E.	B	S.E.					B	S.E.	B	S.E.		
Scotland	7.17	7.00	6.27	6.20	−0.90***	0.07	−0.80**	0.31	21.76%	24.97%	32.79%	35.51%	11.03%***	0.01	10.53%	0.05	50.68%	42.19%
England	7.00	6.71	6.10	6.19	−0.90***	0.07	−0.53**	0.17	23.57%	28.33%	35.81%	34.15%	12.24%***	0.01	5.83%	0.03	51.93%	20.57%
United Kingdom	7.03	6.74	6.15	6.19	−0.88***	0.06	−0.55***	0.16	23.23%	28.05%	35.12%	34.18%	11.88%***	0.01	6.14%*	0.03	51.15%	21.89%
Wales	7.15	7.04	6.47	6.27	−0.68***	0.08	−0.76**	0.29	22.23%	29.22%	32.15%	35.34%	9.92%***	0.01	6.12%	0.05	44.64%	20.94%
Qatar	7.75	7.15	7.08	6.68	−0.67***	0.06	−0.47***	0.04	19.43%	24.48%	25.59%	29.71%	6.16%***	0.01	5.23%***	0.01	31.70%	21.36%
Northern Ireland	7.24	7.23	6.58	6.42	−0.67***	0.09	−0.82***	0.23	20.50%	21.59%	29.09%	32.19%	8.58%***	0.01	10.60%**	0.03	41.86%	49.10%
Ireland	7.36	7.01	6.77	6.60	−0.59***	0.06	−0.41**	0.14	18.07%	22.76%	26.94%	28.87%	8.87%***	0.01	6.11%*	0.02	49.09%	26.85%
Macao	6.64	6.56	6.07	6.08	−0.57***	0.08	−0.48***	0.06	28.64%	29.66%	37.64%	35.58%	9.00%***	0.02	5.93%***	0.01	31.43%	19.99%
United States	7.42	7.16	6.86	6.46	−0.56***	0.06	−0.70***	0.12	19.48%	22.87%	26.55%	34.02%	7.07%***	0.01	11.15%***	0.02	36.27%	48.78%
United Arab Emirates	7.60	7.12	7.12	6.71	−0.48***	0.07	−0.41***	0.07	21.98%	25.82%	27.42%	29.96%	5.44%***	0.01	4.14%***	0.01	24.76%	16.04%
Iceland	7.82	7.40	7.37	6.90	−0.45***	0.06	−0.50	0.28	14.93%	19.27%	17.50%	22.29%	2.58%**	0.01	3.02%	0.04	17.25%	15.70%
France	7.66	7.44	7.22	6.99	−0.44***	0.04	−0.45***	0.13	13.49%	17.92%	19.09%	22.99%	5.61%***	0.01	5.07%*	0.02	41.57%	28.29%
Russia	7.75	7.82	7.33	7.15	−0.42***	0.07	−0.68***	0.19	17.36%	18.36%	22.74%	27.58%	5.39%***	0.01	9.22%**	0.03	31.03%	50.21%
Austria	7.59	7.26	7.21	6.87	−0.38***	0.07	−0.39**	0.12	15.74%	22.02%	20.83%	27.54%	5.09%***	0.01	5.52%**	0.02	32.33%	25.05%
Slovenia	7.19	7.00	6.84	7.02	−0.35***	0.06	0.02	0.22	21.41%	28.04%	26.73%	25.57%	5.32%***	0.01	−2.47%	0.04	24.83%	−8.82%
Chile	7.38	7.08	7.04	6.52	−0.34***	0.07	−0.55	0.31	21.45%	27.87%	25.69%	32.70%	4.24%***	0.01	4.83%	0.05	19.76%	17.35%
Estonia	7.51	7.42	7.18	7.26	−0.33***	0.05	−0.16	0.15	17.15%	22.88%	21.03%	23.06%	3.88%***	0.01	0.18%	0.03	22.60%	0.79%
Germany	7.36	7.30	7.03	6.98	−0.33***	0.06	−0.32**	0.12	18.06%	20.82%	22.95%	24.95%	4.89%***	0.01	4.13%*	0.02	27.07%	19.83%
Netherlands	7.80	8.03	7.47	7.72	−0.33***	0.04	−0.32*	0.13	6.71%	7.72%	8.76%	8.00%	2.05%**	0.01	0.27%	0.02	30.52%	3.56%
Switzerland	7.79	7.58	7.48	7.18	−0.31***	0.06	−0.40***	0.09	11.51%	15.67%	15.56%	20.05%	4.05%***	0.01	4.38%**	0.02	35.19%	27.97%
Finland	7.90	7.84	7.63	7.39	−0.27***	0.05	−0.46*	0.22	11.15%	15.40%	15.15%	17.97%	4.00%***	0.01	2.56%	0.03	35.88%	16.65%
Lithuania	7.88	7.43	7.61	7.53	−0.27***	0.05	0.10	0.30	14.55%	18.63%	17.81%	21.12%	3.26%***	0.01	2.49%	0.05	22.42%	13.35%
Slovakia	7.47	7.02	7.22	6.98	−0.25***	0.05	−0.04	0.54	19.52%	29.18%	22.00%	23.49%	2.47%**	0.01	−5.68%	0.08	12.65%	−19.48%
Costa Rica	8.22	8.11	7.97	7.85	−0.25***	0.05	−0.26	0.14	11.25%	12.38%	14.23%	16.24%	2.97%***	0.01	3.85%	0.02	26.44%	31.10%
Hong Kong	6.53	6.38	6.30	6.26	−0.24***	0.06	−0.12	0.10	27.33%	31.04%	31.02%	32.40%	3.69%**	0.01	1.36%	0.02	13.51%	4.37%
Luxembourg	7.43	7.33	7.20	6.93	−0.23***	0.06	−0.40***	0.07	17.44%	20.42%	20.88%	25.18%	3.45%**	0.01	4.76%***	0.01	19.77%	23.29%
Latvia	7.38	7.15	7.15	7.29	−0.23***	0.05	0.15	0.22	16.84%	21.54%	21.53%	21.76%	4.69%***	0.01	0.22%	0.04	27.82%	1.02%
Portugal	7.38	7.19	7.15	6.87	−0.23***	0.06	−0.33	0.20	17.44%	21.96%	20.83%	26.53%	3.39%***	0.01	4.57%	0.03	19.47%	20.80%
Croatia	7.89	7.97	7.69	7.65	−0.20***	0.05	−0.32*	0.16	13.07%	13.24%	16.92%	18.91%	3.85%***	0.01	5.68%*	0.02	29.43%	42.90%
Mexico	8.28	7.92	8.13	7.49	−0.15**	0.05	−0.43	0.57	11.64%	16.17%	12.18%	18.83%	0.54%	0.01	2.66%	0.08	4.65%	16.43%
Czech Republic	7.06	6.83	6.93	6.49	−0.13*	0.06	−0.34	0.35	24.18%	28.46%	25.22%	29.17%	1.03%	0.01	0.70%	0.06	4.28%	2.48%
Hungary	7.17	7.38	7.11	7.39	−0.06	0.07	0.01	0.26	22.05%	14.10%	22.99%	16.18%	0.94%	0.01	2.08%	0.04	4.27%	14.76%
Spain	7.48	6.92	7.43	6.82	−0.05	0.04	−0.10	0.10	15.24%	23.34%	16.18%	25.75%	0.94%	0.01	2.40%	0.01	6.17%	10.30%
Montenegro	7.77	7.42	7.75	6.64	−0.02	0.05	−0.78**	0.25	18.20%	22.83%	19.20%	32.17%	1.00%	0.01	9.34%*	0.04	5.47%	40.91%
Greece	6.94	6.64	6.99	6.92	0.04	0.05	0.28	0.18	23.76%	28.83%	22.85%	27.09%	−0.90%	0.01	−1.74%	0.03	−3.80%	−6.04%
Italy	6.92	6.63	6.98	6.30	0.07	0.05	−0.34	0.20	24.23%	28.83%	21.64%	32.29%	−2.59%**	0.01	3.46%	0.04	−10.67%	12.00%
Bulgaria																		
Brazil																		
Colombia																		
Japan																		
South Korea																		
Peru																		
Poland																		
Taiwan																		
Thailand																		
Turkey																		
Uruguay																		

Notes: countries are ordered from greater to smaller change in mean life satisfaction among native students in the period 2015–2018. Native students are those born in the country and whose parents were also born in the country. Immigrant students are those born out of the country or whose father and/or mother were born out of the country

Low life satisfaction is defined as rating your life satisfaction with 5 or less in the 0 to 10 life satisfaction scale.

Comparisons involving categories with less than 50 observations were excluded from the analysis due to small sample size.

* p < .05, ** p < .01, and *** p < .001

**Table 6:** Change in students’ mean life satisfaction between 2015 and 2018, by urbanity

	2015					2018				
	<3 K	3-15 K	15-100 K	100 K-1 M	>1 M	<3 K	3-15 K	15-100 K	100 K-1 M	>1 M
England	7.10	7.03	6.92	6.76	6.87	6.15	6.23	6.04	6.08	6.17
United Kingdom	7.16	7.07	6.94	6.86	6.87	6.25	6.26	6.10	6.10	6.18
Northern Ireland	7.42	7.47	7.32	7.13		6.77	6.58	6.57	6.40	
Scotland	7.52	7.13	7.09	7.29		6.40	6.20	6.35	6.14	
Poland	7.34	7.28	7.28	6.85	6.78	6.90	6.64	6.65	6.71	6.89
Russia	8.03	7.84	7.76	7.72	7.56	7.97	7.34	7.18	7.20	7.02
Ireland	7.37	7.28	7.23	7.20	7.33	6.98	6.73	6.66	6.88	6.54
Brazil	7.75	7.75	7.73	7.52	7.28	7.63	7.31	7.16	6.88	6.98
Wales	7.17	7.13	7.09	7.18		6.49	6.36	6.52	6.32	
United States	7.59	7.54	7.35	7.20	7.25	6.89	6.84	6.85	6.61	6.60
Chile	7.23	7.63	7.58	7.24	7.31	6.82	7.43	7.09	6.96	7.03
Japan		6.13	6.75	6.79	6.91		5.92	6.29	6.14	6.20
France	7.72	7.62	7.65	7.58	7.48	7.19	7.19	7.20	7.09	7.42
Luxembourg		7.36	7.40	7.38			7.09	6.95	7.05	
United Arab Emirates	7.64	7.61	7.41	7.28	7.13	7.41	7.17	6.99	6.96	6.57
Qatar	7.71	7.57	7.29	7.38	7.35	6.92	6.92	6.87	6.81	6.79
Iceland	7.71	7.89	7.81	7.76		7.31	7.36	7.40	7.31	
Germany		7.37	7.41	7.23	7.20		7.05	7.02	6.98	7.09
Turkey		6.16	6.07	6.20	6.11		6.01	5.72	5.54	5.58
Netherlands		7.78	7.85	7.88			7.49	7.51	7.44	
Estonia	7.53	7.50	7.56	7.43		7.24	7.03	7.22	7.23	
Slovenia	7.75	7.27	7.10	7.17		7.33	6.78	6.79	6.88	
Finland	7.75	7.89	7.92	7.91		7.58	7.49	7.62	7.70	
Slovakia	7.49	7.65	7.49	7.10		7.34	7.39	7.19	6.94	
Mexico	8.26	8.25	8.37	8.28	8.18	8.11	8.05	8.08	8.16	8.09
Colombia	8.18	8.09	7.99	7.77	7.65	7.78	8.03	7.76	7.59	7.30
Switzerland	7.77	7.80	7.63	7.61		7.45	7.39	7.40	7.27	
Portugal	7.52	7.45	7.31	7.39	7.24	7.61	7.17	7.10	7.09	7.08
Bulgaria	7.47	7.28	7.48	7.38	7.48	7.43	7.18	7.29	7.06	6.94
Costa Rica	8.18	8.26	8.17	8.20		8.16	7.98	7.99	7.72	
Lithuania	7.86	7.92	7.93	7.80		7.83	7.55	7.75	7.44	
Uruguay	8.10	7.70	7.74	7.78	7.54	7.63	7.66	7.57	7.59	7.40
Latvia	7.43	7.35	7.34	7.37		7.04	7.20	7.19	7.15	
Peru	7.45	7.65	7.40	7.39	7.81	7.53	7.29	7.26	7.20	6.91
Czech Republic	6.87	7.18	7.07	7.05	6.88	7.03	6.99	6.94	6.91	6.59
Croatia		7.95	7.94	7.90	7.32		7.71	7.82	7.58	7.42
Austria	7.83	7.58	7.49	7.65	7.27	7.42	7.19	7.36	7.23	6.62
Greece	6.99	6.99	6.97	6.80	6.70	7.28	7.19	6.89	6.92	6.88
Hungary		7.31	7.12	7.30	7.11		7.28	7.06	7.11	7.07
Thailand	7.96	7.87	7.61	7.44	7.65	7.94	7.78	7.58	7.53	7.37
Montenegro		7.93	7.77	7.65			7.65	7.74	7.62	
Taiwan	6.97	6.70	6.61	6.60	6.54	6.50	6.56	6.61	6.54	6.42
Spain	7.55	7.49	7.38	7.41	7.34	7.53	7.39	7.38	7.30	7.18
Italy	7.28	6.83	6.89	6.92	6.82	7.48	6.97	6.95	6.89	6.64
South Korea		6.84	6.34	6.35	6.35		6.32	6.66	6.46	6.51
Hong Kong										
Macao										

	Change									
	<3 K	3-15 K	3-15 K		15-100 K		100 K-1 M		>1 M	
	B	S.E.	B	S.E.	B	S.E.	B	S.E.	B	S.E.
England	−0.95**	0.31	−0.80***	0.17	−0.88***	0.11	−0.69***	0.15	−0.70**	0.23
United Kingdom	−0.91***	0.24	−0.82***	0.13	−0.85***	0.09	−0.76***	0.13	−0.70**	0.22
Northern Ireland	−0.66	0.46	−0.89***	0.20	−0.74***	0.16	−0.73***	0.17		
Scotland	−1.12***	0.16	−0.93***	0.14	−0.74***	0.12	−1.15***	0.15		
Poland	−0.44***	0.12	−0.64***	0.13	−0.63***	0.11	−0.13	0.13	0.10	0.22
Russia	−0.06	0.21	−0.49***	0.14	−0.58***	0.11	−0.51***	0.12	−0.54***	0.11
Ireland	−0.39**	0.12	−0.56***	0.11	−0.57***	0.12	−0.32*	0.15	−0.79***	0.12
Brazil	−0.12	0.28	−0.44***	0.13	−0.57***	0.09	−0.64***	0.07	−0.30**	0.11
Wales	−0.68**	0.22	−0.78***	0.16	−0.56***	0.11	−0.87***	0.10		
United States	−0.70**	0.22	−0.69***	0.15	−0.51***	0.11	−0.59***	0.11	−0.65**	0.20
Chile	−0.41	0.45	−0.19	0.19	−0.49***	0.11	−0.27**	0.09	−0.27*	0.11
Japan			−0.20	0.28	−0.46***	0.10	−0.65***	0.07	−0.71***	0.11
France	−0.53	0.31	−0.42***	0.08	−0.45***	0.05	−0.49***	0.12	−0.06	0.15
Luxembourg			−0.28***	0.08	−0.45***	0.10	−0.33***	0.07		
United Arab Emirates	−0.22	0.17	−0.44***	0.12	−0.42***	0.12	−0.32***	0.09	−0.56***	0.11
Qatar	−0.78***	0.18	−0.66***	0.08	−0.41***	0.09	−0.56***	0.06	−0.55***	0.09
Iceland	−0.40**	0.15	−0.53***	0.13	−0.41***	0.10	−0.46***	0.09		
Germany			−0.32**	0.11	−0.39***	0.08	−0.24*	0.12	−0.11	0.24
Turkey			−0.15	0.38	−0.36*	0.16	−0.66***	0.18	−0.53***	0.11
Netherlands			−0.29*	0.15	−0.34***	0.05	−0.44***	0.08		
Estonia	−0.29*	0.12	−0.47***	0.11	−0.34***	0.09	−0.20*	0.08		
Slovenia	−0.42	0.23	−0.49***	0.11	−0.32***	0.08	−0.29*	0.12		
Finland	−0.17	0.15	−0.39***	0.07	−0.30***	0.06	−0.21**	0.08		
Slovakia	−0.15	0.16	−0.26	0.14	−0.29***	0.07	−0.17	0.21		
Mexico	−0.15	0.17	−0.20	0.14	−0.29***	0.09	−0.13	0.07	−0.09	0.07
Colombia	−0.40	0.20	−0.06	0.23	−0.23	0.13	−0.18	0.11	−0.35***	0.07
Switzerland	−0.32	0.19	−0.41***	0.08	−0.22*	0.11	−0.34**	0.13		
Portugal	0.09	0.31	−0.28**	0.09	−0.22***	0.06	−0.30**	0.10	−0.16	0.28
Bulgaria	−0.04	0.40	−0.10	0.14	−0.19	0.11	−0.32*	0.13	−0.55***	0.10
Costa Rica	−0.02	0.09	−0.29**	0.10	−0.19*	0.09	−0.48**	0.16		
Lithuania	−0.03	0.10	−0.37***	0.09	−0.18	0.10	−0.36***	0.08		
Uruguay	−0.47*	0.22	−0.04	0.12	−0.17	0.10	−0.18	0.17	−0.14	0.10
Latvia	−0.39*	0.16	−0.16	0.09	−0.14	0.09	−0.22*	0.09		
Peru	0.08	0.13	−0.36***	0.09	−0.14	0.11	−0.19	0.13	−0.91***	0.27
Czech Republic	0.17	0.28	−0.19	0.14	−0.13	0.10	−0.14	0.13	−0.29	0.19
Croatia			−0.24*	0.10	−0.13	0.08	−0.32***	0.09	0.10	0.17
Austria	−0.41	0.27	−0.39***	0.10	−0.12	0.13	−0.42***	0.12	−0.64***	0.12
Greece	0.29	0.22	0.20	0.12	−0.08	0.10	0.12	0.11	0.18	0.15
Hungary			−0.03	0.15	−0.05	0.11	−0.20	0.12	−0.04	0.15
Thailand	−0.03	0.17	−0.09	0.11	−0.03	0.10	0.09	0.14	−0.28	0.22
Montenegro			−0.27*	0.13	−0.03	0.07	−0.03	0.09		
Taiwan	−0.47	0.41	−0.13	0.16	0.00	0.07	−0.06	0.10	−0.12	0.07
Spain	−0.02	0.18	−0.10	0.08	0.00	0.08	−0.11	0.08	−0.16	0.12
Italy	0.20*	0.08	0.14	0.14	0.06	0.08	−0.03	0.15	−0.18	0.23
South Korea			−0.52	0.36	0.32*	0.15	0.12	0.09	0.16	0.09
Hong Kong										
Macao										

Notes: countries are ordered from greater to smaller change in mean life satisfaction between 2015 and 2018 in schools located in municipalities with a population between 15,000 and 100,000 people

Comparisons involving categories with less than 50 observations were excluded from the analysis due to small sample size.

* p < .05, ** p < .01, and *** p < .001

**Table 7:** Change in the proportion of students reporting low life satisfaction between 2015 and 2018, by urbanity

	2015					2018					Absolute Change	
	<3 K	3-15 K	15-100 K	100 K-1 M	>1 M	<3 K	3-15 K	15-100 K	100 K-1 M	>1 M	<3 K *Q Q*	
											B	S.E.
England	19.22%	21.90%	21.90%	28.67%	25.30%	36.75%	32.11%	34.54%	34.52%	35.07%	17.53%**	0.05
United Kingdom	19.19%	21.67%	21.67%	27.02%	25.23%	34.44%	32.10%	34.03%	34.15%	34.98%	15.25%***	0.04
Poland	20.78%	20.83%	20.83%	25.91%	28.25%	25.99%	28.63%	28.83%	26.18%	25.94%	5.21%**	0.02
Wales	21.47%	22.59%	22.59%	23.39%		27.09%	30.63%	31.50%	33.29%		5.62%	0.04
N. Ireland	17.50%	16.23%	16.23%	20.72%		26.16%	30.65%	28.18%	29.19%		8.66%	0.09
Scotland	16.85%	21.41%	21.41%	20.07%		35.51%	33.43%	31.18%	33.25%		18.66%***	0.03
Ireland	16.72%	19.57%	19.57%	19.22%	17.31%	23.04%	27.55%	28.04%	26.16%	30.25%	6.31%**	0.02
Russia	14.99%	16.21%	16.21%	17.18%	18.20%	16.86%	22.03%	24.22%	23.74%	25.01%	1.87%	0.03
United States	18.14%	18.69%	18.69%	22.12%	22.49%	26.05%	28.12%	25.66%	30.87%	29.05%	7.91%*	0.04
Chile	23.85%	17.66%	17.66%	24.08%	21.63%	28.68%	21.15%	24.14%	26.15%	24.92%	4.83%	0.11
Germany		17.79%	17.79%	16.45%	17.74%		19.33%	21.15%	22.15%	19.86%		
Japan		31.64%	31.64%	27.14%	25.11%		45.04%	34.46%	38.21%	36.52%		
Brazil	16.25%	16.03%	16.03%	18.04%	21.03%	15.02%	20.57%	22.43%	25.59%	26.03%	−1.23%	0.03
Luxembourg		20.41%	20.41%	17.14%			23.23%	25.67%	22.16%			
Slovenia	15.73%	21.26%	21.26%	21.84%		18.38%	28.18%	28.13%	23.96%		2.65%	0.04
United Arab Emirates	21.10%	22.92%	22.92%	24.53%	24.38%	26.17%	26.79%	27.31%	26.11%	30.88%	5.07%	0.03
France	13.83%	14.56%	14.56%	13.59%	13.18%	20.70%	19.69%	18.80%	21.87%	16.13%	6.87%	0.06
Estonia	17.69%	17.81%	17.81%	18.02%		20.77%	23.20%	20.92%	19.49%		3.08%	0.02
Turkey		40.72%	40.72%	41.41%	40.69%		40.76%	44.91%	46.59%	45.29%		
Finland	13.97%	11.18%	11.18%	10.65%		15.73%	16.25%	14.77%	14.72%		1.76%	0.03
Latvia	16.65%	16.82%	16.82%	17.65%		22.54%	21.37%	20.24%	21.34%		5.90%*	0.02
Slovakia	20.58%	16.51%	16.51%	23.73%		22.61%	18.78%	22.17%	22.89%		2.03%	0.03
Croatia		12.97%	12.97%	11.81%	20.09%		17.32%	16.03%	17.26%	20.45%		
Netherlands		9.26%	9.26%	6.65%			7.45%	8.66%	9.74%			
Iceland	14.93%	13.44%	13.44%	15.72%		17.96%	18.06%	17.21%	17.18%		3.03%	0.02
Thailand	18.07%	15.13%	15.13%	19.51%	14.91%	16.73%	20.14%	20.84%	20.48%	18.79%	−1.34%	0.02
Qatar	18.78%	20.27%	20.27%	21.66%	22.19%	30.11%	28.19%	26.06%	27.32%	26.58%	11.33%***	0.03
Colombia	13.60%	15.32%	15.32%	17.67%	19.57%	15.73%	14.32%	17.73%	19.12%	22.03%	2.13%	0.02
Portugal	14.97%	17.24%	17.24%	15.69%	17.60%	17.35%	19.97%	20.36%	19.96%	21.90%	2.38%	0.05
Costa Rica	12.27%	9.52%	9.52%	12.21%		14.84%	13.78%	13.42%	15.45%		2.56%*	0.01
Switzerland	14.04%	11.50%	11.50%	13.94%		16.92%	17.66%	15.51%	17.94%		2.88%	0.04
Hungary		20.39%	20.39%	18.85%	22.17%		20.98%	23.77%	23.01%	21.52%		
Austria	13.24%	15.88%	15.88%	15.28%	20.57%	17.26%	21.21%	19.31%	20.35%	30.44%	4.02%	0.04
Greece	25.26%	24.56%	24.56%	24.92%	25.32%	21.40%	20.89%	23.96%	24.08%	23.26%	−3.86%	0.04
Lithuania	15.31%	13.67%	13.67%	14.56%		16.38%	18.85%	15.26%	19.11%		1.07%	0.02
Bulgaria	22.68%	23.01%	23.01%	21.50%	19.17%	17.16%	23.97%	22.03%	24.49%	24.99%	−5.52%	0.05
Montenegro		15.49%	15.49%	19.38%			19.85%	19.13%	20.87%			
Mexico	14.75%	12.71%	12.71%	11.01%	10.48%	9.50%	12.06%	10.97%	10.85%	12.29%	−5.25%**	0.02
Peru	22.88%	18.90%	18.90%	21.18%	16.48%	14.71%	20.79%	22.50%	21.78%	24.15%	−8.17%***	0.02
Taiwan	27.96%	32.13%	32.13%	31.93%	30.82%	34.84%	33.58%	32.38%	31.09%	32.22%	6.89%	0.07
Czech Republic	31.02%	22.21%	22.21%	22.94%	24.00%	25.14%	24.45%	24.43%	25.60%	29.23%	−5.88%	0.05
Uruguay	13.57%	17.69%	17.69%	14.10%	18.24%	17.42%	16.34%	16.52%	17.34%	18.67%	3.85%	0.03
Spain	14.15%	15.26%	15.26%	15.61%	16.25%	17.33%	16.50%	17.20%	17.20%	18.75%	3.18%	0.03
Italy	17.74%	24.50%	24.50%	22.06%	22.84%	16.99%	22.58%	21.48%	22.18%	26.08%	−0.74%	0.03
South Korea		29.96%	29.96%	34.88%	33.69%		35.99%	30.52%	33.45%	32.53%		
Hong Kong												
Macao												

	Absolute Change								Relative Change				
	3-15 K Q Q		15-100 K Q Q		100 K-1 M Q Q		>1 M *Q Q*		<3 K	3-15 K	15-100 K	100 K-1 M	>1 M
	B	S.E.	B	S.E.	B	S.E.	B	S.E.					
England	10.22%***	0.03	10.53%***	0.02	5.85%*	0.03	9.77%*	0.04	91.19%	46.66%	48.08%	20.40%	38.61%
United Kingdom	10.43%***	0.02	10.25%***	0.02	7.13%***	0.02	9.75%*	0.04	79.44%	48.15%	47.33%	26.39%	38.66%
Poland	7.80%***	0.02	9.87%***	0.02	0.27%	0.02	−2.30%	0.04	25.10%	37.45%	47.39%	1.04%	−8.16%
Wales	8.04%**	0.02	9.25%***	0.02	9.90%***	0.02			26.17%	35.57%	40.96%	42.31%	
N. Ireland	14.42%***	0.03	8.74%**	0.03	8.47%**	0.03			49.49%	88.83%	53.86%	40.86%	
Scotland	12.01%***	0.02	7.81%***	0.02	13.19%***	0.03			110.76%	56.11%	36.46%	65.72%	
Ireland	7.98%***	0.02	7.76%***	0.02	6.95%**	0.03	12.94%***	0.02	37.76%	40.77%	39.65%	36.16%	74.75%
Russia	5.82%**	0.02	7.58%***	0.02	6.56%***	0.02	6.81%***	0.02	12.46%	35.91%	46.75%	38.16%	37.43%
United States	9.43%***	0.03	7.11%***	0.02	8.75%***	0.02	6.56%*	0.03	43.61%	50.44%	38.06%	39.55%	29.15%
Chile	3.50%	0.03	6.27%***	0.02	2.07%	0.02	3.29%*	0.02	20.25%	19.81%	35.52%	8.60%	15.24%
Germany	1.54%	0.02	6.14%***	0.02	5.70%*	0.02	2.12%	0.04		8.67%	34.49%	34.67%	11.95%
Japan	13.40%**	0.05	6.04%**	0.02	11.07%***	0.01	11.41%***	0.02		42.36%	19.08%	40.81%	45.46%
Brazil	4.54%*	0.02	5.97%***	0.01	7.55%***	0.01	5.01%**	0.02	−7.58%	28.31%	37.24%	41.82%	23.80%
Luxembourg	2.82%*	0.01	5.85%***	0.02	5.02%***	0.01				13.80%	28.65%	29.26%	
Slovenia	6.92%***	0.02	5.83%***	0.01	2.11%	0.02			16.86%	32.57%	27.43%	9.67%	
United Arab Emirates	3.87%*	0.02	4.91%**	0.02	1.58%	0.01	6.50%***	0.02	24.01%	16.88%	21.42%	6.44%	26.65%
France	5.13%**	0.02	4.76%***	0.01	8.28%***	0.02	2.95%	0.03	49.67%	35.27%	32.67%	60.95%	22.42%
Estonia	5.39%**	0.02	4.40%***	0.01	1.47%	0.01			17.40%	30.24%	24.68%	8.16%	
Turkey	0.04%	0.05	4.06%	0.02	5.18%*	0.03	4.60%**	0.02		0.10%	9.96%	12.52%	11.30%
Finland	5.07%***	0.01	3.92%***	0.01	4.07%**	0.01			12.61%	45.34%	35.10%	38.20%	
Latvia	4.54%**	0.02	3.71%*	0.02	3.69%*	0.01			35.43%	27.02%	22.07%	20.91%	
Slovakia	2.27%	0.02	3.26%**	0.01	−0.84%	0.03			9.84%	13.77%	19.78%	−3.53%	
Croatia	4.35%**	0.02	2.82%**	0.01	5.46%***	0.01	0.35%	0.03		33.58%	21.75%	46.20%	1.76%
Netherlands	−1.81%	0.02	2.72%***	0.01	3.09%*	0.01				−19.59%	29.38%	46.45%	
Iceland	4.62%*	0.02	2.54%	0.02	1.46%	0.02			20.27%	34.42%	18.90%	9.31%	
Thailand	5.00%**	0.02	2.33%	0.02	0.97%	0.03	3.88%	0.03	−7.43%	33.06%	15.38%	4.98%	26.02%
Qatar	7.92%***	0.01	2.29%	0.01	5.66%***	0.01	4.40%**	0.01	60.35%	39.05%	11.27%	26.15%	19.82%
Colombia	−1.00%	0.03	2.12%	0.02	1.45%	0.02	2.45%	0.01	15.62%	−6.53%	13.83%	8.23%	12.54%
Portugal	2.73%	0.02	2.03%	0.01	4.27%*	0.02	4.30%	0.06	15.88%	15.82%	11.80%	27.23%	24.45%
Costa Rica	4.26%***	0.01	1.94%	0.01	3.24%	0.02			20.89%	44.79%	20.37%	26.55%	
Switzerland	6.16%***	0.01	1.55%	0.02	4.01%	0.02			20.48%	53.58%	13.49%	28.74%	
Hungary	0.58%	0.02	1.45%	0.02	4.16%	0.02	−0.65%	0.03		2.86%	7.10%	22.08%	−2.93%
Austria	5.33%**	0.02	1.43%	0.02	5.07%**	0.02	9.87%***	0.02	30.39%	33.56%	8.99%	33.21%	48.01%
Greece	−3.67%	0.02	1.21%	0.02	−0.84%	0.02	−2.06%	0.03	−15.29%	−14.93%	4.95%	−3.36%	−8.12%
Lithuania	5.18%***	0.01	1.21%	0.01	4.54%***	0.01			6.97%	37.90%	8.86%	31.21%	
Bulgaria	0.97%	0.02	1.14%	0.02	2.99%	0.02	5.82%***	0.01	−24.34%	4.20%	4.94%	13.92%	30.35%
Montenegro	4.35%*	0.02	1.12%	0.01	1.49%	0.01				28.10%	7.25%	7.66%	
Mexico	−0.65%	0.02	0.74%	0.01	−0.16%	0.01	1.80%	0.01	−35.57%	−5.09%	5.85%	−1.44%	17.20%
Peru	1.90%	0.01	0.71%	0.02	0.59%	0.02	7.67%	0.04	−35.72%	10.03%	3.78%	2.80%	46.53%
Taiwan	1.45%	0.03	0.60%	0.02	−0.85%	0.02	1.40%	0.01	24.63%	4.53%	1.87%	−2.66%	4.53%
Czech Republic	2.24%	0.02	0.35%	0.02	2.66%	0.03	5.23%	0.03	−18.96%	10.09%	1.58%	11.59%	21.79%
Uruguay	−1.35%	0.02	0.05%	0.02	3.24%	0.02	0.43%	0.02	28.39%	−7.66%	0.27%	22.96%	2.35%
Spain	1.24%	0.01	0.03%	0.01	1.59%	0.01	2.50%	0.02	22.48%	8.15%	0.23%	10.17%	15.36%
Italy	−1.92%	0.02	−3.46%**	0.01	0.12%	0.02	3.25%	0.04	−4.19%	−7.82%	−14.12%	0.52%	14.23%
South Korea	6.03%	0.07	−3.98%	0.03	−1.43%	0.02	−1.16%	0.02		20.13%	−13.29%	−4.10%	−3.45%
Hong Kong													
Macao													

Notes: countries are ordered from greater to smaller absolute change in the % of students reporting low satisfaction (i.e. 5 points or less in the 0 to 10 life satisfaction scale) between 2015 and 2018 in schools located in municipalities with a population between 15,000 and 100,000 people

Comparisons involving categories with less than 50 observations were excluded from the analysis due to small sample size.

* p < .05, ** p < .01, and *** p < .001
