# Supplementary material for: A Global Decline in Adolescents’ Subjective Well-Being: a Comparative Study Exploring Patterns of Change in the Life Satisfaction of 15-Year-Old Students in 46 Countries
Source: Child Indic Res. Author manuscript; Available in PMC 2021 Sep 17. (PMC7611680; doi:10.1007/s12187-020-09788-8)
Supplement: Appendix [file EMS133113-supplement-Appendix.pdf]

Appendix

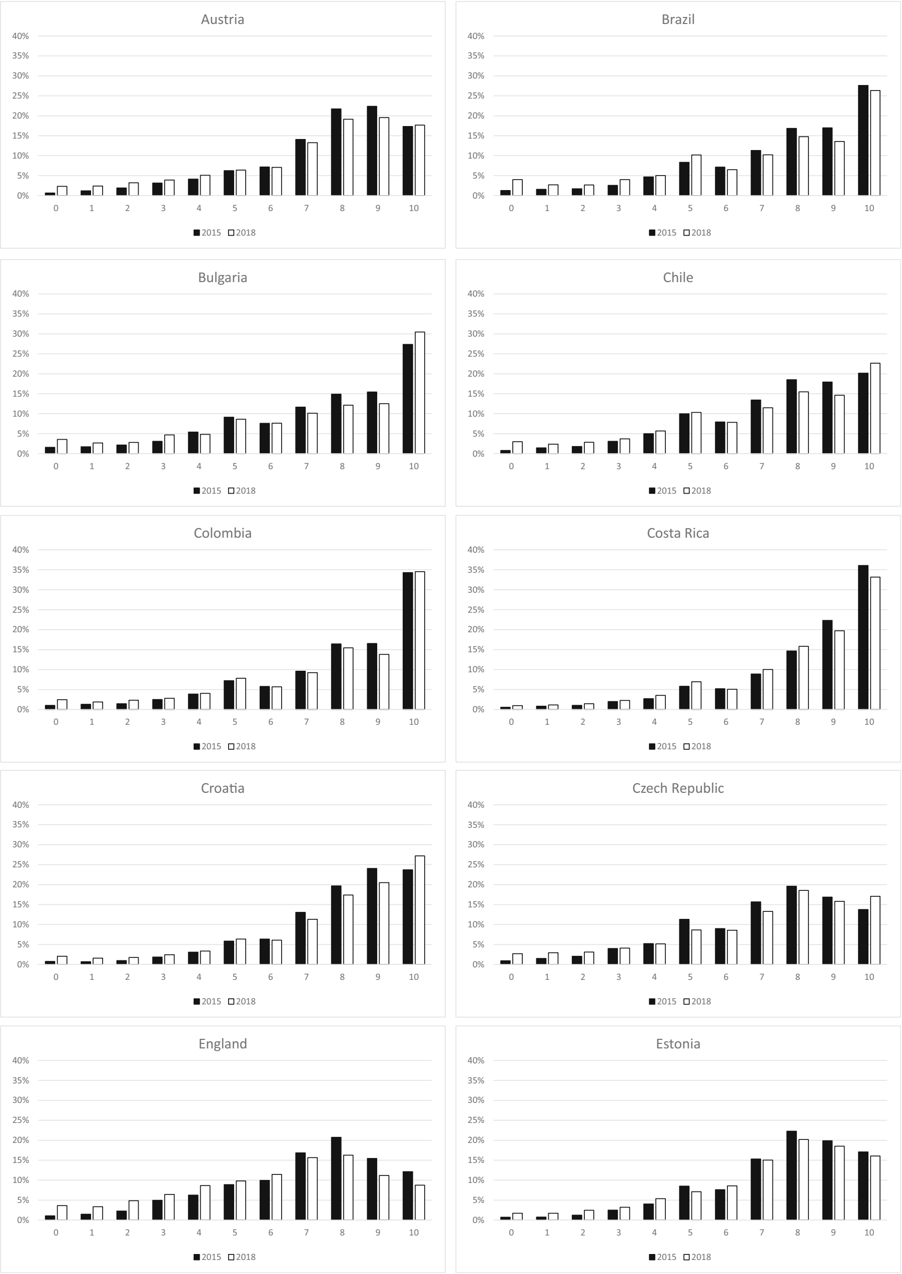

Fig. 1 Students' responses in the life satisfaction scale in 2015 and 2018, by country (I)

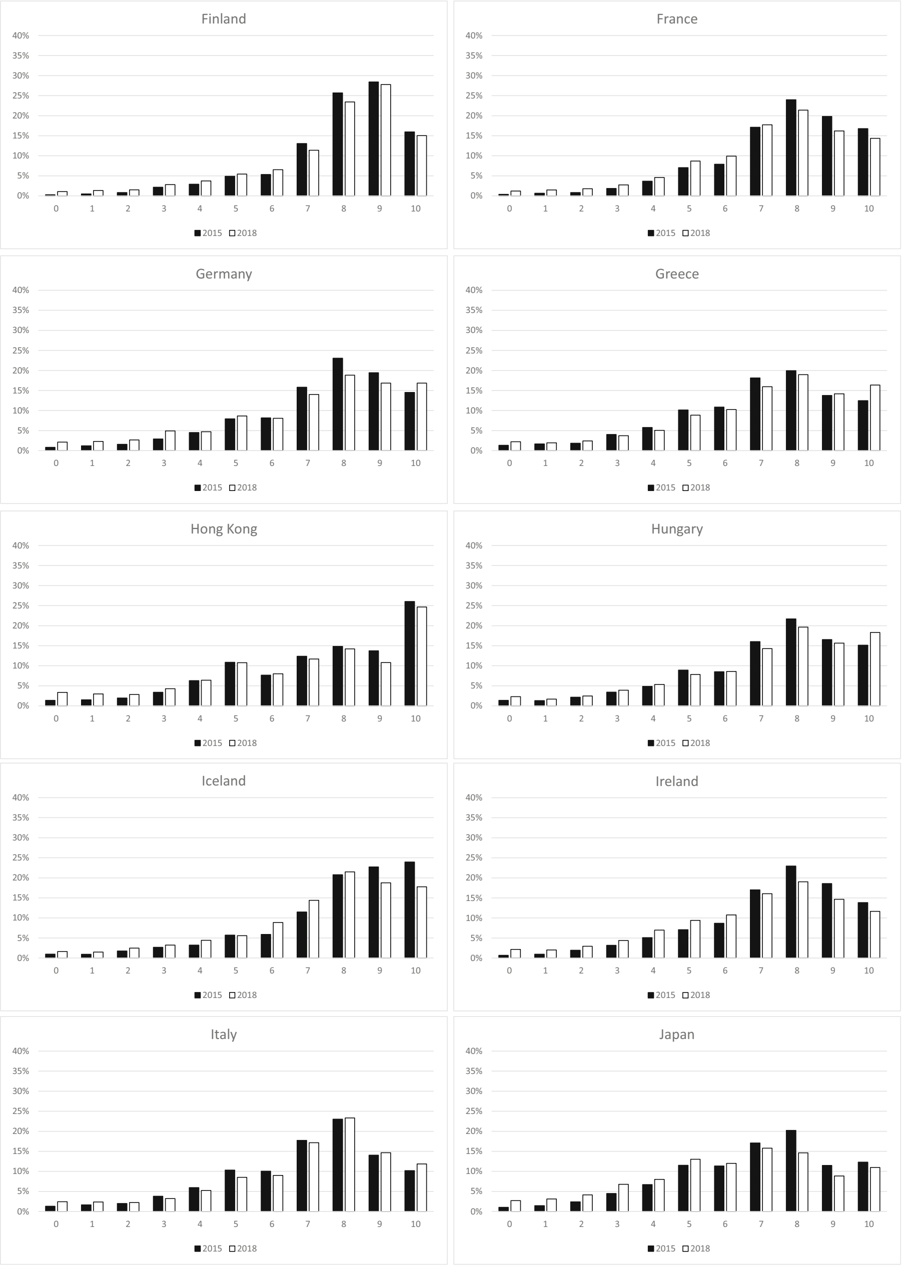

Fig. 2 Students' responses in the life satisfaction scale in 2015 and 2018, by country (II)

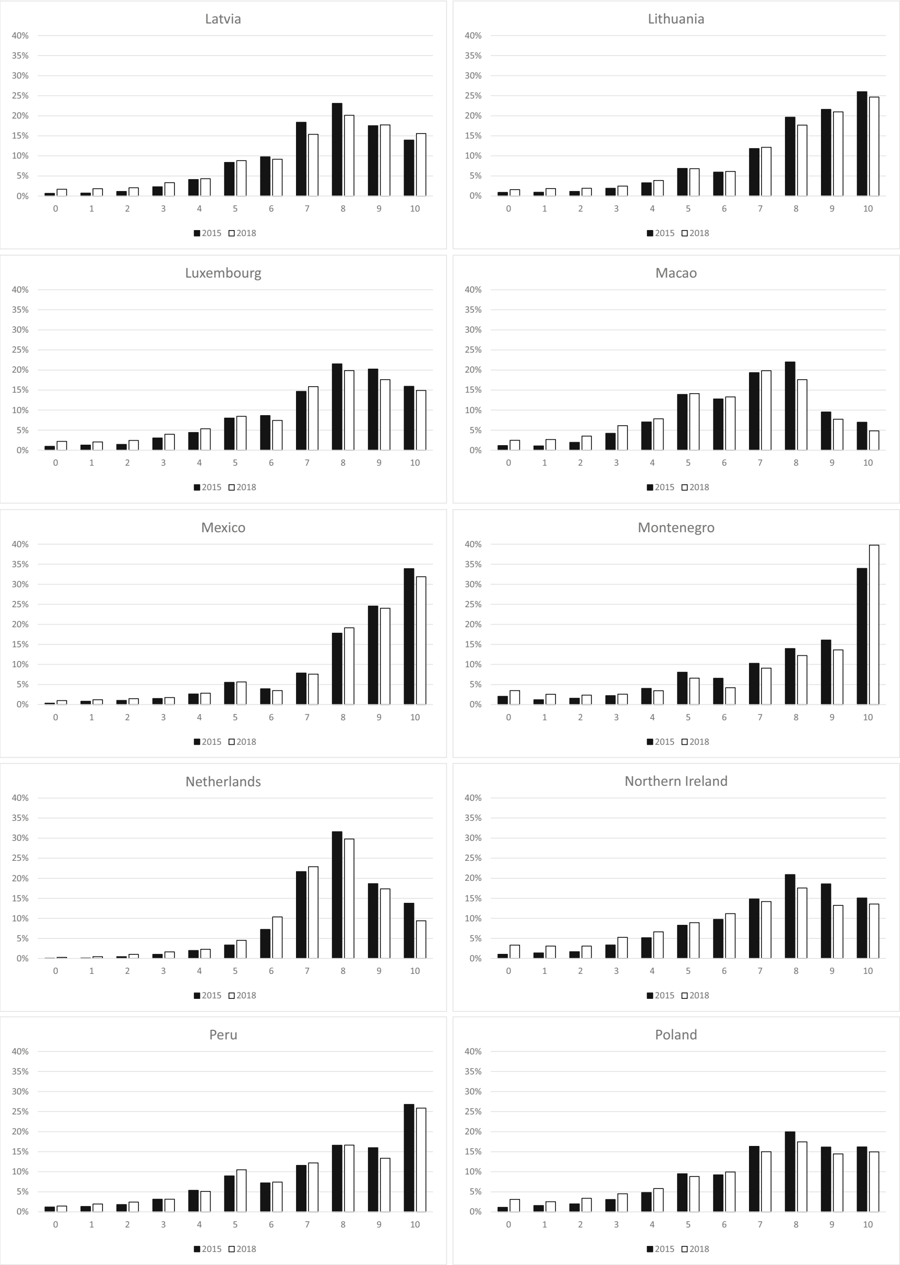

Fig. 3 Students' responses in the life satisfaction scale in 2015 and 2018, by country (III)

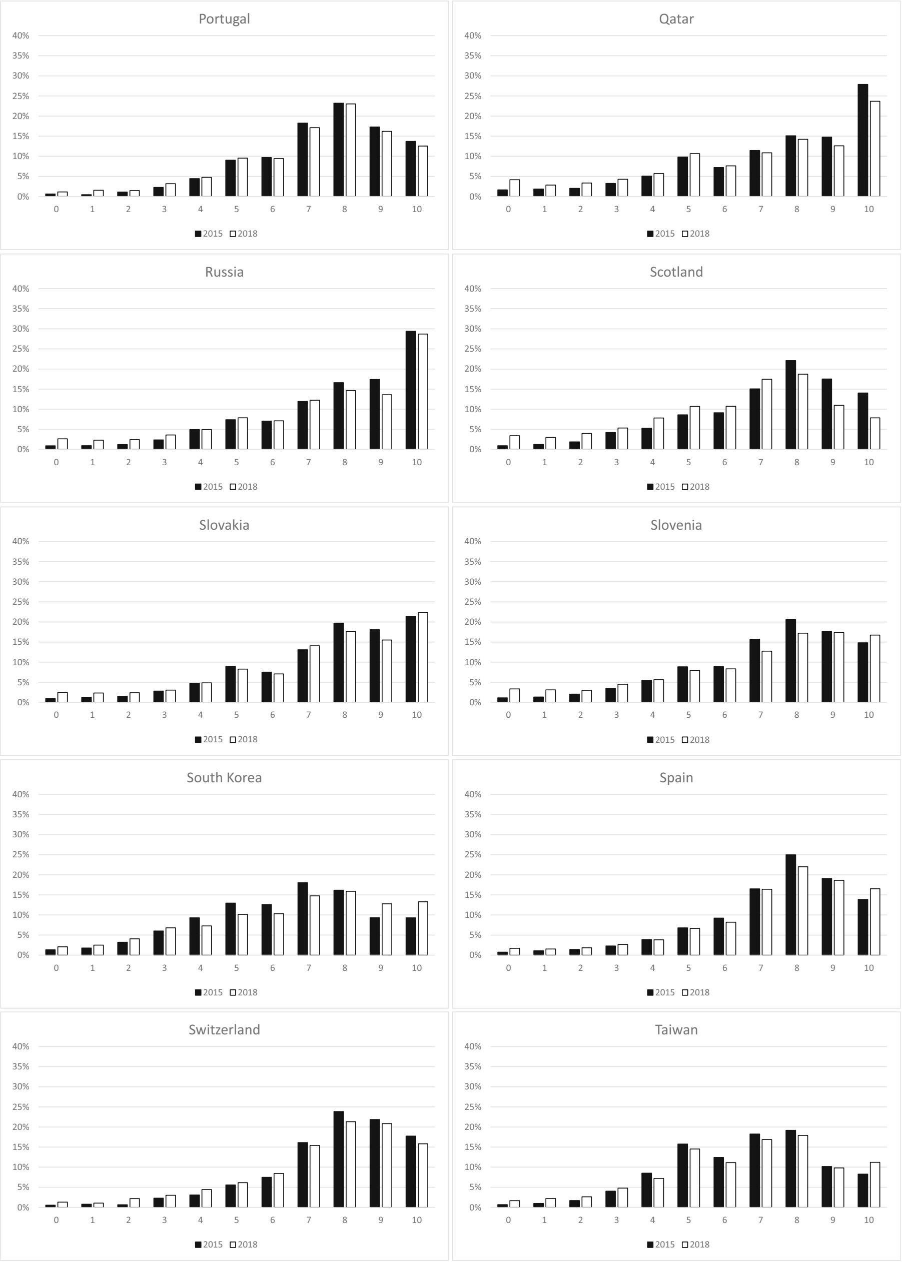

Fig. 4 Students' responses in the life satisfaction scale in 2015 and 2018, by country (IV)

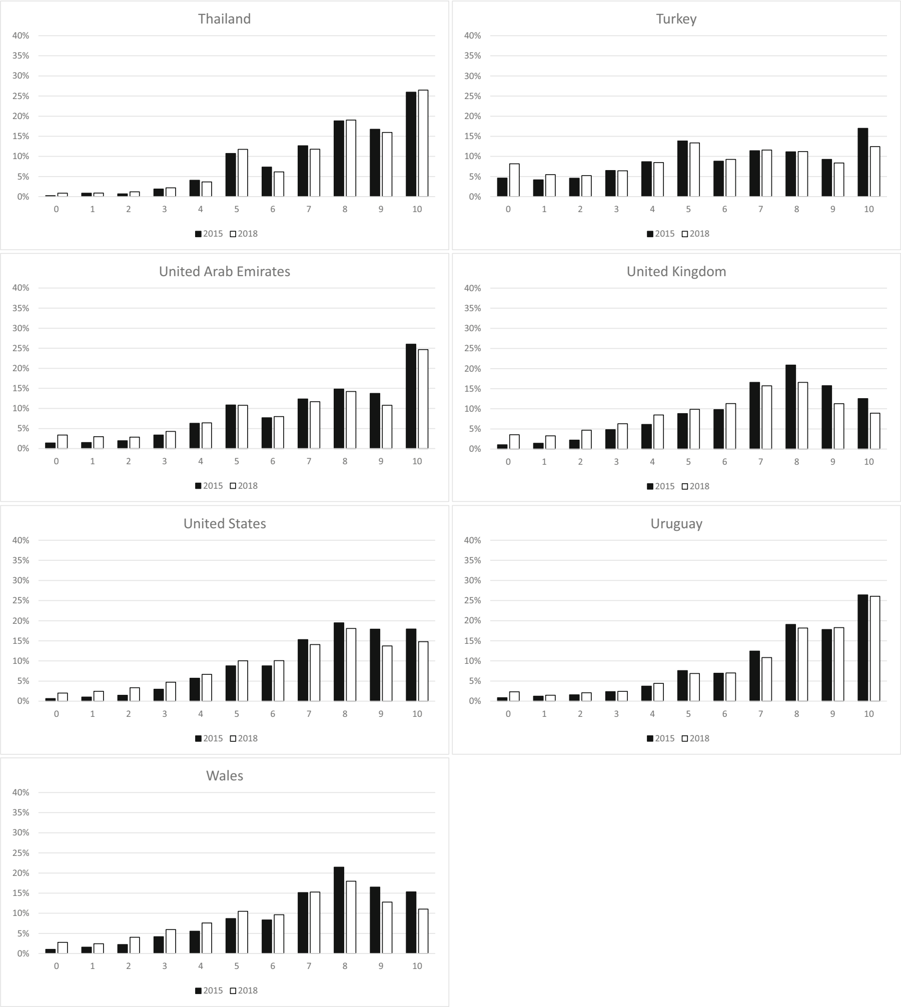

Fig. 5 Students' responses in the life satisfaction scale in 2015 and 2018, by country (V)

Open Access This article is licensed under a Creative Commons Attribution 4.0 International License, which permits use, sharing, adaptation, distribution and reproduction in any medium or format, as long as you give appropriate credit to the original author(s) and the source, provide a link to the Creative Commons licence, and indicate if changes were made. The images or other third party material in this article are included in the article's Creative Commons licence, unless indicated otherwise in a credit line to the material. If material is not included in the article's Creative Commons licence and your intended use is not permitted by statutory regulation or exceeds the permitted use, you will need to obtain permission directly from the copyright holder. To view a copy of this licence, visit http://creativecommons.org/licenses/by/4.0/.
